# Lifetime risk of developing diabetes in Chinese people with normoglycemia or prediabetes: A modeling study

**DOI:** 10.1371/journal.pmed.1004045

**Published:** 2022-07-21

**Authors:** Xinge Zhang, Hongjiang Wu, Baoqi Fan, Mai Shi, Eric S. H. Lau, Aimin Yang, Elaine Chow, Alice P. S. Kong, Juliana C. N. Chan, Ronald C. W. Ma, Andrea O. Y. Luk

**Affiliations:** 1 Department of Medicine and Therapeutics, The Chinese University of Hong Kong, Hong Kong, Special Administrative Region, People’s Republic of China; 2 Hong Kong Institute of Diabetes and Obesity, The Chinese University of Hong Kong, Hong Kong, Special Administrative Region, People’s Republic of China; 3 Li Ka Shing Institute of Health Sciences, The Chinese University of Hong Kong, Hong Kong, Special Administrative Region, People’s Republic of China; University of Cambridge, UNITED KINGDOM

## Abstract

**Background:**

Little is known about the lifetime risk of progression to diabetes in the Asian population. We determined remaining lifetime risk of diabetes and life years spent with diabetes in Chinese people with normoglycemia and prediabetes.

**Methods and findings:**

Using territory-wide diabetes surveillance data curated from electronic medical records of Hong Kong Hospital Authority (HA), we conducted a population-based cohort study in 2,608,973 individuals followed from 2001 to 2019. Prediabetes and diabetes were identified based on laboratory measurements, diagnostic codes, and medication records. Remaining lifetime risk and life years spent with diabetes were estimated using Monte Carlo simulations with state transition probabilities based on a Markov chain model. Validations were performed using several sensitivity analyses and modified survival analysis. External replication was performed using the China Health and Retirement Longitudinal Survey (CHARLS) cohort (2010 to 2015).

The expected remaining lifetime risk of developing diabetes was 88.0 (95% confidence intervals: 87.2, 88.7)% for people with prediabetes and 65.9 (65.8, 65.9)% for people with normoglycemia at age 20 years. A 20-year-old person with prediabetes would live with diabetes for 32.5 (32.0, 33.1) years or 51.6 (50.8, 52.3)% of remaining life years, whereas a person with normoglycemia at 20 years would live 12.7 (12.7, 12.7) years with diabetes or 18.4 (18.4, 18.5)% of remaining life years. Women had a higher expected remaining lifetime risk and longer life years with diabetes compared to men. Results are subjected to possible selection bias as only people who undertook routine or opportunistic screening were included.

**Conclusions:**

These findings suggest that Hong Kong, an economically developed city in Asia, is confronted with huge challenge of high lifetime risk of diabetes and long life years spent with diabetes, especially in people with prediabetes. Effective public health policies and targeted interventions for preventing progression to diabetes are urgently needed.

## Abstract 2

Xinge Zhang and colleagues estimate remaining lifetime risk of diabetes and life years spent with diabetes in Chinese people with normoglycaemia and prediabetes.

## Author summary

**Why was this study done?:**

- Lifetime risk is a valuable estimate for raising public awareness and education.
- The majority of studies reporting lifetime risk of diabetes were conducted in non-Asian.
- Little is known about remaining lifetime risks of diabetes and life years spent with diabetes in East Asian population.

**What did the researchers do and find?:**

- Using a territory-wide database of over 2.6 million people living in Hong Kong, we estimated lifetime risk of diabetes and life years spent with diabetes.
- According to our estimates, up to two-thirds of Chinese people with normoglycemia and 9 in 10 of Chinese people with prediabetes at age 20 years would develop diabetes during their remaining lifetime.
- People with normoglycemia and prediabetes at age 20 years were expected to live with diabetes for an average of 13 and 33 years, or 18% and 52% of their remaining lifetime, respectively.

**What do these findings mean?:**

- The extremely high expected lifetime risk of diabetes in Chinese people is alarming, especially in those with prediabetes.
- Our findings give added impetus to policy makers and healthcare stakeholders to develop and implement strategies aiming to prevent progression of diabetes in our region.
- Our findings are subjected to possible selection bias as only people who undertook routine or opportunistic screening were included.

## Introduction

Prediabetes, typically defined as blood glucose concentrations higher than normal but lower than diabetes thresholds, is a high-risk state for diabetes development [1]. Globally, approximately 374 million people are living with prediabetes with a further increase of over 20% anticipated in the next 10 years [2]. Intensive lifestyle modification has been shown to prevent or delay the onset of diabetes in high-risk individuals, but large-scale implementation of preventive programs remains difficult to achieve even in high-income economies [3,4]. Effective communication with policy makers and other stakeholders on health and societal impact of diabetes is needed to stimulate public health responses and motivate change.

Lifetime risk is defined as the probability that a person who is currently free of a condition will acquire it at some time during his/her life span [5]. Owing to its nature of being easier to comprehend for conveying diabetes risk to people compared with other measures such as incidence, prevalence, or relative risk, lifetime risk is a valuable estimate for raising public awareness and education. Several studies have reported the lifetime risk of diabetes, but most of these were conducted in countries of predominantly white population [6–9]. In an early study in the United States of America, lifetime risks of diabetes were estimated to be 33% and 39% for men and women born in 2000, respectively [6–8]. Lifetime risk estimates are sensitive to population differences in propensity for prediabetes and diabetes and mortality rates. For example, expected lifetime risks of diabetes were 24% in Denmark [10], 38% in Australia [9], and 56% to 65% in India [11]. Previous studies have suggested that East Asians have a higher incidence of type 2 diabetes [12–20]. In this context, we aimed to determine the remaining lifetime risk of progression from normoglycemia and prediabetes to diabetes for Chinese people, using data from the territory-wide Hong Kong Diabetes Surveillance Database (HKDSD).

## Methods

### Data sources and study population

The Hong Kong Hospital Authority (HA) governs all public hospitals and the majority of community-based primary care clinics to provide about 90% of healthcare services for Hong Kong residents [21]. In 2000, the Hong Kong HA adopted an electronic medical record system that captures diagnostic and procedure codes, laboratory tests, medication prescription, and linked vital status (time and causes of death) longitudinally. The HKDSD comprises clinical information of all individuals who have ever had at least 1 glycemic measurement including fasting plasma glucose (FPG), random plasma glucose, glycated hemoglobin (HbA1c), and 2-hour oral glucose tolerance test (OGTT) at any health facilities in Hong Kong HA. All individuals who attended routine screening for diabetes or had opportuntistic testing during clinical encounters were included. Detailed information has been reported elsewhere [13,22].

From January 2000 to December 2019, 4,089,903 people have been included in the HKDSD. To avoid misclassification of stress-induced and gestational hyperglycemia, all records of FPG during hospital admissions and all glycemic measurements within 24 to 40 gestational week were excluded from the analysis. We limited our analyses to 2001 to 2019 to avoid bias from incomplete case records of diabetes in the first year of establishment of the electronic medical record system. Finally, 2,608,973 people in Hong Kong who had measured FPG, HbA1c, and/or 2-hour OGTT for at least once from 2001 to 2019 were included in this analysis. In this cohort, 21.2% had 1 glycemic measurement, and 11.9%, 6.4%, 5.3%, and 55.2% had 2, 3, 4, and 5 or more glycemic measurements, respectively. Observation time was less than 1 year in 29.7%, between 1 and 5 years in 19.9%, and 5 years or longer in 50.4%. S1 Fig shows the changes in the distribution of different glycemic assessments for the first 5 tests. The majority of people repeated the same glycemic assessment at their next test. S2 Fig shows the proportions of people who had at least 1 glycemic measurement to the midyear population of Hong Kong across calendar period [23]. The analysis was planned in September 2021, and additional analyses were conducted in March 2022 in response to suggestions of journal reviewers. The study was approved by the Joint Chinese University of Hong Kong–New Territories East Cluster Clinical Research Ethics Committee. Individual informed consent was not obtained because all records in the HKDSD were anonymized at the time of access, and individual informed consent was deemed not required by the Ethics Committee because of the retrospective nature of the analysis.

### Definition of prediabetes and diabetes

Prediabetes and diabetes were identified according to the American Diabetes Association (ADA) criteria [24]. Prediabetes was defined as meeting the following criteria before the diagnosis of diabetes: FPG ≥5.6 and <7.0 mmol/L, or HbA1c ≥39 and <48 mmol/mol (≥5.7 and <6.5%), or 2-hour OGTT ≥7.8 and <11.1 mmol/L, in any one available measurement. People who met one or more of the following criteria were identified as having diabetes: (1) diagnosis of diabetes by clinicians based on the International Classification of Diseases-Ninth Revision (ICD-9) code 250.xx, and/or the revised edition of the International Classification of Primary Care, World Organization of National Colleges, Academics, and Academic Associations of General Practitioners/Family Physicians code T89 or code T90; (2) prescription of non-insulin glucose-lowering drugs; (3) prescription of insulin continuously for ≥28 days; (4) FPG ≥7.0 mmol/L; (5) HbA1c ≥48 mmol/mol (6.5%); and (6) 2-hour OGTT ≥11.1 mmol/L. Diabetes types were not differentiated. Normoglycemia referred to all remaining records that did not fulfill the diagnostic criteria of prediabetes or diabetes.

### Statistical analyses

The standard Kaplan–Meier method adjusted for competing risk of death using the cumulative incidence function was applied to examine short-term cumulative risk of progression from prediabetes to diabetes over a 19-year period from 2001 to 2019 stratified by age at baseline and by sex. People were categorized into 4 age groups: <20 years, ≥20 and <40 years, ≥40 and <60 years, and ≥60 years. For comparison, we also performed analyses of progression from normoglycemia to diabetes. For progression from prediabetes to diabetes, the baseline was set as the time of onset of prediabetes. For progression from normoglycemia to diabetes, the baseline was set as the entry of the individual in HKDSD, i.e., at the first glycemic measurement.

To estimate the remaining lifetime risk, we constructed a discrete-time Markov chain model to simulate possible transitions of an individual of a given age and sex through the 4 mutually exclusive states: “Normoglycemia,” “Prediabetes,” “Diabetes,” and “Death” (S3 Fig), where death was treated as the absorbing states (once entered, cannot leave). We adopted the mstate R package [25] to estimate the transition probabilities using the data from the HKDSD, details are shown in S1 Text. As very few people survived over 110 years, we set the maximum age for estimating transition probabilities as 110 years. Next, we used the transition probabilities to simulate the glycemic progression of 3 million individuals of a specified baseline sex from the age 0 through to 110 years, with a cycle length of 1 year. Using this simulated cohort, we calculated the remaining lifetime risk of progression to diabetes at an index age as the proportion of people who would eventually develop diabetes among people who had prediabetes or normoglycemia at the index age. We also calculated the length of life that a person is expected to live without and with diabetes as the mean number of years from index age to onset age of diabetes and the mean number of years from the onset age of diabetes to the age of death, respectively. To evaluate the performance of the simulated cohort, we plotted standard Kaplan–Meier survival curves, adjusting for competing risk of death, for predicted cumulative risk of progression from prediabetes to diabetes using the simulated cohort, stratified by onset age of prediabetes and sex, and compared them with the curves of short-term risk generated from the HKDSD.

We examined changes in expected lifetime risks of diabetes over calendar time. We split the HKDSD data into 2 periods, 2001 to 2009 and 2010 to 2019, and reestimated lifetime risks of diabetes in each period. We examined birth cohort effects on expected lifetime risks of diabetes. We estimated the lifetime risk of diabetes for each birth cohort from 1900 to 1980 using the modified Kaplan–Meier method. Incidence rates of diabetes and mortality rates at each age were required for lifetime risk estimation. However, the HKDSD had data for up to 19 years only for each birth cohort (e.g., for people born in 1950, data were available for ages 51 to 69 years only). Therefore, we developed age–period–cohort models to extrapolate the rates for ages without available data. We used natural cubic splines to fit the effects of age, period, and birth cohorts. Knots were placed at the maximum and minimum values and at each inflection point identified manually by examining a plot.

Several sensitivity analyses were conducted as follows: (1) In the primary analysis, the type of diabetes was not differentiated. On the assumption that people with type 1 diabetes would usually have developed diabetes by age 20 years, we repeated the estimations by considering only individuals aged 20 years or above in the simulation in whom type 2 diabetes predominate; (2) Considering the effect of diagnosis performance of different measurements of prediabetes [26], we redefined prediabetes according to FPG only and HbA1c only, then repeated the estimations. As only 1.4% of people in the HKDSD had 2-hour OGTT, we did not address impaired glucose tolerance (IGT) as a separate state model. (3) Alternative approaches, the modified Kaplan–Meier method developed by the Framingham study group [27] and Sullivan life table method [28,29], were used to estimate the remaining lifetime risk, life years spent with and without diabetes, and proportions of remaining life years with diabetes. Details are given in S2 and S3 Text. (4) To evaluate potential effect of follow-up duration, we reestimated the lifetime risk of diabetes restricting the analysis to people who were observed for at least 5 years in the HKDSD.

### Replication analysis

We replicated the analysis in the China Health and Retirement Longitudinal Survey (CHARLS) cohort [30]. In brief, CHARLS recruited community residents aged 45 to 90 years between June 2011 and March 2012 across 28 provinces and 150 counties/districts covering both urban and rural areas in mainland China using multistage probability sampling method. Out of 12,740 households approached, 10,257 responded with response rate of 80.5%. A total of 17,708 individuals underwent the survey, among whom 11,532 had blood tests for FPG and HbA1c. Between 2011 and 2015, 522 were recorded dead. In 2015, 7,430 returned for repeated blood glucose tests. Therefore, a total of 7,952 individuals were included in the replication analysis. In CHARLS, prediabetes was defined as FPG ≥5.6 and <7.0 mmol/L, and/or HbA1c ≥39 and <48 mmol/mol (≥5.7 and <6.5%), and without self-reported history of diabetes or use of glucose-lowering drugs. Diabetes was defined as self-reported history of diabetes, use of glucose-lowering drugs, FPG ≥7.0 mmol/L, and/or HbA1c≥48 mmol/mol (6.5%). Owing to the limited number of people aged over 90 years in CHARLS, we calculated the remaining lifetime risk of diabetes before 90 years old for people with normoglycemia or prediabetes at age 45 years. The transition probabilities of death from each glycemia status in HKDSD and CHARLS are shown in S4 Fig.

All analyses were conducted using R software, version 4.0.3 (R Foundation for Statistical Computing, Vienna, Austria).

## Results

Of 2,608,973 people (54.2% females) included in this study, 1,014,795 and 830,870 individuals had ever been identified to have prediabetes and diabetes, respectively (S1 Table). Clinical characteristics of people included in this study at baseline are shown in S2 Table.

### Short-term cumulative risk of progression from prediabetes to diabetes

During 5,767,846 person-years of follow-up in 1,014,795 people with prediabetes, 250,175 people developed diabetes at a crude incidence of 43.4 per 1,000 person-years. Over the 19-year period, the probabilities of progression from prediabetes to diabetes after adjusting for competing risk of death were 23.9% (95% confidence intervals: 18.7%, 29.2%) for youth aged <20 years, 46.8% (45.4%, 48.2%) for people aged ≥20 and <40 years, 60.4% (59.9%, 61.0%) for people aged ≥40 and <60 years, and 45.1% (44.8%, 45.4%) for those aged ≥60 years (S5 Fig). Boys (aged <20 years) with prediabetes had a lower risk of progression than girls, but the sex difference in risks reversed in people aged ≥20 years (S6 and S7 Figs).

During 11,412,840 person-years follow-up in 1,521,199 people with normal glucose at baseline, 122,705 developed diabetes at a crude incidence of 10.8 per 1,000 person-years. The 19-year risks of progression from normoglycemia to diabetes after adjusting for competing risk of death were 5.6% (4.8%, 6.3%) for youth aged <20 years, 12.9% (12.4%, 13.5%) for people aged ≥20 and <40 years, 24.1% (23.7%, 24.6%) for people aged ≥40 and <60 years, and 21.1% (20.7%, 21.5%) for those aged ≥60 years (S5 Fig). Men with normoglycemia had a higher risk of developing diabetes than women across all age groups (S6 and S7 Figs).

### Expected remaining lifetime risk of diabetes

**Fig 1:**
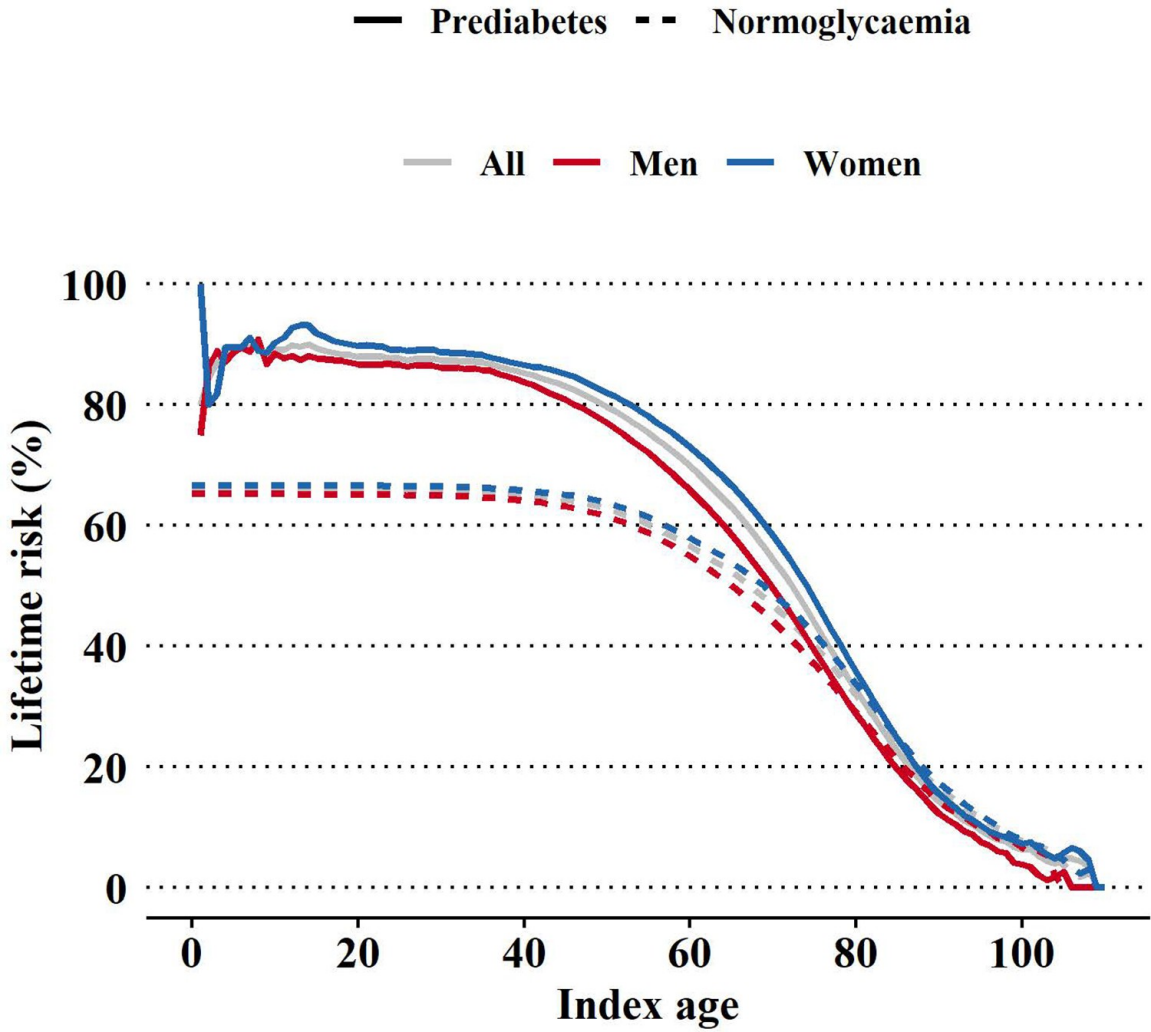
Expected remaining lifetime risk of progression to diabetes across index age in men and women by glycemic status.

We determined the transition probabilities between normoglycemia, prediabetes, diabetes, and death at the next age (S8 Fig). In the simulation of 3 million individuals, 1,743,856 developed prediabetes and 1,979,219 developed diabetes during their life spans (S3 Table). The expected remaining lifetime risk of developing diabetes was 88.0 (87.2, 88.7)% for people with prediabetes at age 20 years and 65.9 (65.8, 65.9)% for people with normoglycemia at age 20 years (Fig 1 and Table 1). The number of people who developed prediabetes before 20 years was small, resulting in unstable estimates in expected remaining lifetime risk for youth. Overall, the expected remaining lifetime risk of progression to diabetes decreased with increasing index age. Women had a higher expected remaining lifetime risk of progressing from both prediabetes and normoglycemia to diabetes compared to men.

**Table 1:** Expected remaining lifetime risks of diabetes, remaining life years living with and without diabetes, and proportions of remaining life years spent with diabetes for men and women with prediabetes and normoglycemia at age 20, 40, and 60 years.

	All people		Men		Women	
	From prediabetes	From normoglycemia	From prediabetes	From normoglycemia	From prediabetes	From normoglycemia
Remaining lifetime risk of diabetes, %						
Age 20 years	88.0 (87.2, 88.7)	65.9 (65.8, 65.9)	86.8 (85.7, 87.8)	65.1 (65.0, 65.2)	89.7 (88.6, 90.9)	66.6 (66.5, 66.6)
Age 40 years	85.2 (84.9, 85.5)	65.1 (65.0, 65.1)	83.7 (83.3, 84.1)	64.2 (64.1, 64.3)	86.6 (86.2, 87.0)	65.8 (65.7, 65.9)
Age 60 years	69.9 (69.7, 70.0)	56.6 (56.6, 56.7)	65.9 (65.7, 66.1)	54.9 (54.8, 55.0)	73.0 (72.9, 73.2)	58.0 (57.9, 58.1)
Remaining life years living with diabetes						
Age 20 years	32.5 (32.0, 33.1)	12.7 (12.7, 12.7)	30.3 (29.7, 31.0)	12.0 (11.9, 12.0)	35.8 (35.0, 36.7)	13.3 (13.2, 13.3)
Age 40 years	24.3 (24.1, 24.4)	12.0 (12.0, 12.0)	22.3 (22.1, 22.5)	11.2 (11.2, 11.3)	26.1 (25.9, 26.3)	12.6 (12.6, 12.6)
Age 60 years	12.1 (12.0, 12.1)	7.8 (7.8, 7.9)	10.3 (10.2, 10.3)	7.1 (7.1, 7.1)	13.5 (13.4, 13.5)	8.4 (8.4, 8.5)
Remaining life years without diabetes						
Age 20 years	27.6 (26.1, 29.1)	53.9 (53.9, 53.9)	26.1 (24.1, 28.0)	52.0 (52.0, 52.0)	29.3 (27.0, 31.6)	55.5 (55.5, 55.5)
Age 40 years	18.7 (18.6, 18.8)	35.8 (35.8, 35.8)	17.5 (17.4, 17.7)	34.0 (34.0, 34.0)	19.8 (19.6, 20.0)	37.3 (37.3, 37.4)
Age 60 years	14.4 (14.4, 14.4)	21.6 (21.6, 21.6)	13.6 (13.5, 13.6)	20.1 (20.1, 20.1)	15.1 (15.0, 15.1)	22.8 (22.8, 22.8)
Proportions of remaining life years spent with diabetes, %						
Age 20 years	51.6 (50.8, 52.3)	18.4 (18.4, 18.5)	50.1 (49.1, 51.0)	18.1 (18.0, 18.1)	53.8 (52.7, 55.0)	18.7 (18.7, 18.8)
Age 40 years	53.7 (53.5, 54.0)	24.7 (24.7, 24.7)	52.8 (52.4, 53.2)	24.5 (24.4, 24.5)	54.6 (54.2, 55.0)	24.9 (24.8, 24.9)
Age 60 years	42.2 (42.1, 42.3)	26.1 (26.0, 26.1)	39.2 (39.0, 39.4)	25.4 (25.3, 25.5)	44.7 (44.5, 44.8)	26.6 (26.5, 26.7)

Figures in brackets represent 95% confidence intervals.

### Number of years expected living with and without diabetes

**Fig 2:**
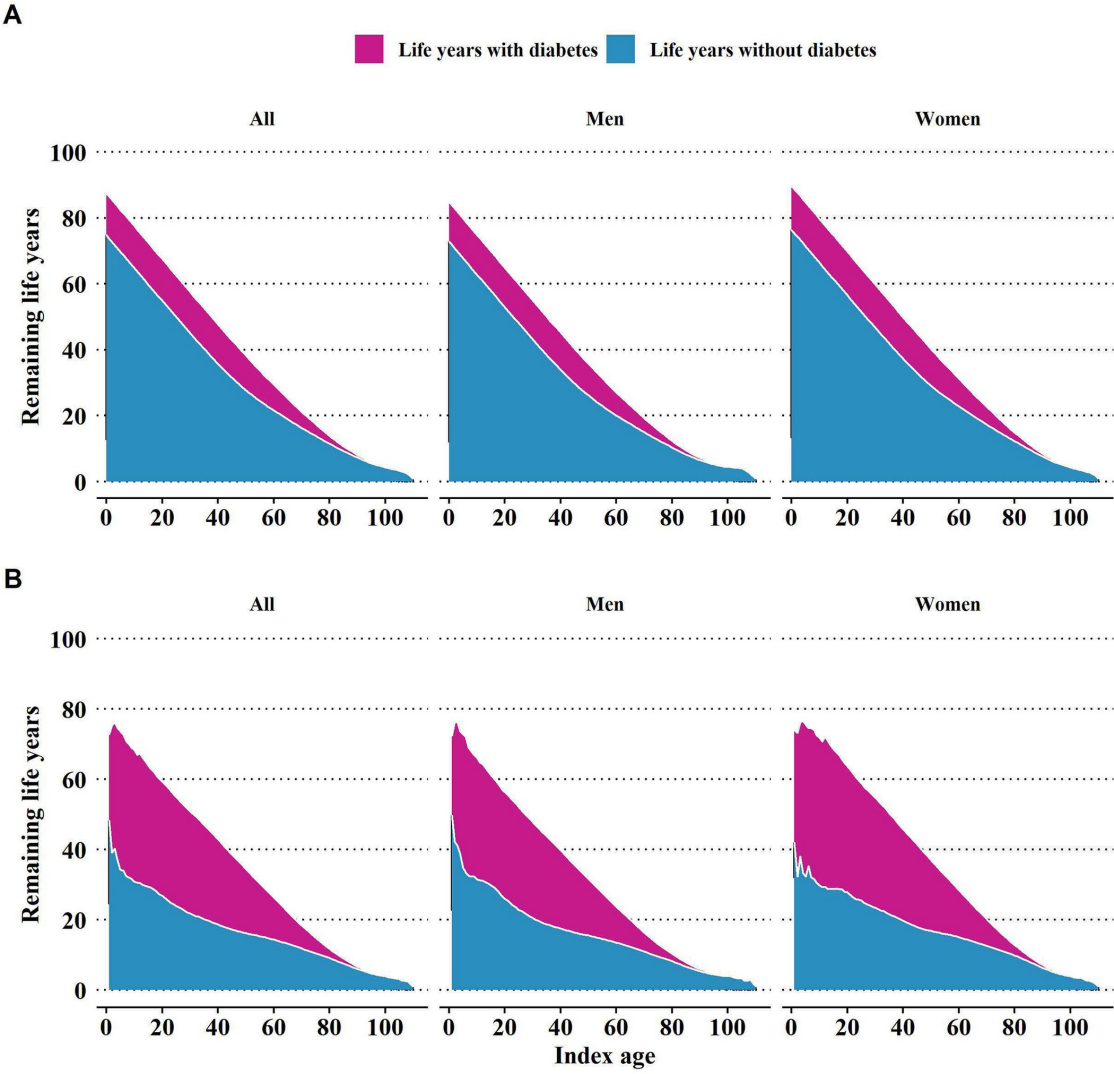
Expected remaining life years with and without diabetes after index age in men and women according to glycemia status. **(A)** People with normoglycemia. **(B)** People with prediabetes.

A 20-year-old person with normoglycemia would be expected to live 12.7 (12.7, 12.7) years with diabetes, which accounts for 18.4 (18.4, 18.5)% of remaining life years (Table 1). In contrast, a 20-year-old person with prediabetes would live an average of 32.5 (32.0, 33.1) years with diabetes, or 51.6 (50.8, 52.3)% of remaining life years (Table 1). The proportion of remaining life years spent living with diabetes was higher in people with prediabetes than their counterparts with normoglycemia. Compared with men, women would live more years with diabetes but have a lower proportion of remaining life years spent with diabetes (Fig 2 and S4–S6 Tables).

### Period and birth cohort effects on expected remaining lifetime risks of diabetes

The expected lifetime risks of diabetes estimated from data in the period of 2001 to 2009 were higher than risks estimated from the period of 2010 to 2019 across all ages (S9 Fig). As an illustration, the expected lifetime risk of diabetes was 89.6 (87.5, 91.7)% for a 20-year-old person with prediabetes and 65.4 (65.0, 65.8)% for a 20-year-old person with normoglycemia in 2001 to 2009. The corresponding estimates were 85.1 (84.2, 85.9)% and 59.8 (59.5, 60.0) for their peers in 2010 to 2019.

The expected lifetime risk of diabetes at age 20 years increased with recency of birth year from 1900 to 1980 (S10 Fig). The expected lifetime risks of diabetes were 64.2 (64.0, 64.4)%, 76.5 (76.4, 76.5)%, and 79.9 (79.9, 79.9)% for a 20-year-old person with prediabetes born in 1900, 1940, and 1980, respectively.

### Verification and validation of generated cohort and sensitivity analyses

The short-term probabilities of progression from prediabetes to diabetes as estimated in the simulated cohort was similar to the observed rates in the HKDSD for most age groups except for people aged <20 years (S11 Fig). This indicated an accurate fitting performance of the simulated cohort except for simulation in youth. When the simulated cohort was limited to people aged 20 years or above, the expected remaining lifetime risks, remaining life years with and without diabetes, and proportions of remaining life years with diabetes were similar to the results of the primary analyses (Tables 2 and S4–S6 and S12 and S13 Figs), indicating that type 1 diabetes had little effect on the estimations. When prediabetes was redefined using either FPG or HbA1c, the estimations did not deviate from those of the primary analyses (Tables 2 and S4–S6 and S14–S17 Figs). Remaining lifetime risks calculated using the modified Kaplan–Meier method (Table 2 and S18 Fig) and length of life expected to be living with diabetes estimated by the Sullivan life table (Tables 2 and S4–S6 and S19 Fig) were similar to those from the primary results. When we restricted the analysis to people who were followed at least 5 years, the estimations were largely unchanged compared with those of the primary analyses (Table 2 and S20 Fig).

**Table 2:** Expected remaining lifetime risks of progression from prediabetes and normoglycemia to diabetes, expressed as percentages, at age 20, 40, and 60 years estimated in primary analyses and sensitivity analyses.

	All people		Men		Women	
	From prediabetes	From normoglycemia	From prediabetes	From normoglycemia	From prediabetes	From normoglycemia
Estimates in the primary results						
Age 20 years	88.0 (87.2, 88.7)	65.9 (65.8, 65.9)	86.8 (85.7, 87.8)	65.1 (65.0, 65.2)	89.7 (88.6, 90.9)	66.6 (66.5, 66.6)
Age 40 years	85.2 (84.9, 85.5)	65.1 (65.0, 65.1)	83.7 (83.3, 84.1)	64.2 (64.1, 64.3)	86.6 (86.2, 87.0)	65.8 (65.7, 65.9)
Age 60 years	69.9 (69.7, 70.0)	56.6 (56.6, 56.7)	65.9 (65.7, 66.1)	54.9 (54.8, 55.0)	73.0 (72.9, 73.2)	58.0 (57.9, 58.1)
Simulation from age 20 years onward						
Age 21 years*	88.2 (85.5, 90.8)	65.9 (65.9, 66.0)	87.4 (83.6, 91.1)	65.1 (65.0, 65.1)	89.1 (85.4, 92.8)	66.6 (66.5, 66.7)
Age 40 years	85.3 (85.0, 85.6)	65.1 (65.0, 65.1)	83.6 (83.2, 84.1)	64.1 (64.1, 64.2)	86.8 (86.4, 87.2)	65.9 (65.8, 65.9)
Age 60 years	69.7 (69.6, 69.9)	56.7 (56.6, 56.8)	65.7 (65.5, 65.9)	54.9 (54.8, 55.0)	72.9 (72.7, 73.1)	58.1 (58.0, 58.2)
Prediabetes was defined by FPG only						
Age 20 years	92.3 (91.3, 93.3)	66.1 (66.1, 66.2)	91.5 (90.2, 92.8)	65.5 (65.4, 65.6)	93.6 (92.1, 95.0)	66.7 (66.6, 66.7)
Age 40 years	89.1 (88.8, 89.4)	65.4 (65.4, 65.5)	87.4 (86.9, 87.9)	64.7 (64.6, 64.7)	90.9 (90.5, 91.4)	66.0 (66.0, 66.1)
Age 60 years	74.8 (74.7, 75.0)	57.1 (57.1, 57.2)	70.9 (70.6, 71.1)	55.5 (55.4, 55.6)	78.2 (78.0, 78.4)	58.5 (58.4, 58.5)
Prediabetes was defined by HbA1c only						
Age 20 years	89.7 (88.8, 90.7)	65.2 (65.1, 65.2)	88.0 (86.7, 89.3)	64.7 (64.6, 64.8)	92.4 (91.1, 93.8)	65.5 (65.5, 65.6)
Age 40 years	86.1 (85.7, 86.5)	64.5 (64.4, 64.5)	84.9 (84.3, 85.5)	63.9 (63.9, 64.0)	87.2 (86.7, 87.7)	64.9 (64.9, 65.0)
Age 60 years	69.6 (69.4, 69.8)	57.0 (56.9, 57.1)	66.3 (66.0, 66.6)	55.5 (55.4, 55.6)	72.2 (71.9, 72.4)	58.2 (58.1, 58.3)
The modified Kaplan–Meier method						
Age 20 years	87.1 (86.5, 87.8)	58.1 (58.0, 58.3)	85.4 (84.5, 86.2)	58.3 (58.0, 58.5)	89.2 (88.1, 90.2)	58.8 (58.6, 59.1)
Age 40 years	83.4 (83.1, 83.7)	57.5 (57.4, 57.7)	82.0 (81.5, 82.5)	57.7 (57.5, 58.0)	85.1 (84.7, 85.5)	58.1 (57.9, 58.4)
Age 60 years	68.2 (68.0, 68.5)	49.5 (49.3, 49.7)	65.1 (64.8, 65.4)	48.0 (47.8, 48.3)	71.8 (71.5, 72.1)	51.3 (51.1, 51.6)
Restricting to people with 5 years or longer of follow-up						
Age 20 years	88.3 (86.3, 90.2)	65.8 (65.6, 65.9)	85.5 (82.6, 88.3)	64.7 (64.5, 64.9)	92.0 (89.5, 94.6)	66.6 (66.5, 66.8)
Age 40 years	85.6 (84.9, 86.3)	64.9 (64.8, 65.1)	83.6 (82.5, 84.7)	63.8 (63.6, 64.0)	87.3 (86.4, 88.2)	65.9 (65.7, 66.1)
Age 60 years	70.1 (69.8, 70.5)	56.2 (56.0, 56.4)	66.0 (65.5, 66.6)	54.1 (53.8, 54.3)	73.4 (72.9, 73.8)	57.8 (57.6, 58.1)

*People were all assumed to have normoglycemia at age 20 years when we simulated from age 20 years onward. Under this assumption, no one would have prediabetes at age 20 years; hence, estimations are provided for age 21 years.

FPG, fasting plasma glucose; HbA1c, glycated hemoglobin.

We replicated the analysis in the CHARLS cohort and compared the estimates against those generated in the HKDSD. The expected lifetime risks of progression from prediabetes to diabetes from age 45 years to 90 years were 75.9 (73.0, 78.8)% in the CHARLS cohort and 81.6 (81.1, 82.1)% in the HKDSD, with a between-population difference of 5.7 (5.6, 5.8)%. The expected lifetime risks of progression from normoglycemia to diabetes from age 45 years to 90 years were 54.8 (54.7, 54.8)% in the CHARLS cohort and 58.5 (58.4, 58.5)% in the HKDSD, with a between-population difference of 3.7 (3.6, 3.8)% (S21 Fig and S7 Table). The CHARLS cohort had a higher mortality rate than the HKDSD (S4 Fig), and the difference in survival could contribute to differences in expected lifetime risks of diabetes between these cohorts. To explore the effect of mortality, we reestimated lifetime risks for the HKDSD using mortality rates of the CHARLS cohort. When mortality rates of the CHARLS cohort were applied to the HKDSD, the expected lifetime risks of diabetes in the HKDSD were 78.9 (78.3, 79.5)% and 54.8 (54.8, 54.9)% for progression from prediabetes and normoglycemia, respectively, and the differences in risk estimates were narrowed to 3.0 (2.9, 3.1)% and 0.1 (0.0, 0.1)% compared with the CHARLS cohort (S22 Fig and S7 Table).

## Discussion

We estimated remaining lifetime risks of progression to diabetes and life years spent with diabetes among Chinese people with prediabetes and normoglycemia in Hong Kong. According to our estimates, two-thirds of people with normoglycemia at age 20 years would develop diabetes during their remaining lifetime. This proportion was increased to 90% if prediabetes was already present at this early age. People with normoglycemia and prediabetes at age 20 years were expected to live with diabetes for an average of 13 and 33 years, or 18% and 52% of their remaining lifetime, respectively. Women had a higher expected remaining lifetime risk of diabetes and longer life years spent with diabetes than men. The expected lifetime risks of diabetes of the population as a whole decreased during the past 2 decades. Across birth cohort from 1900 to 1980, the expected lifetime risks of diabetes at age 20 years increased with recency of birth year. Our estimations were based on a territory-wide database of over 2.6 million people and were robust to sensitivity analyses using different assumptions and modeling techniques. However, these findings were subjected to selection bias as only people who undertook routine or opportunistic screening were included.

The lifetime risk of diabetes estimated in Hong Kong appeared to be greater than that in countries with a predominantly white demographic. Using a population-based cohort of 10,050 adults followed for 15 years, a study in the Netherlands reported an expected remaining lifetime risk of 74% and 31% in people with and without prediabetes at age 45 years, respectively [31]. In the USA, using data from 598,216 adults collected from the National Health Interview Survey linked to the National Death Index, the expected remaining lifetime risk of diabetes was 40% for men and 40% for women aged 20 years for the period 2000–2011 [8]. A study in Australia in which diabetes progression was determined from a cohort of 5,842 individuals followed between 2000 and 2005, the expected remaining lifetime risk of diabetes was 38% from the age of 25 years [9], similar to findings in the USA. In contrast, we found that the average Hong Kong Chinese people aged 20 years with normoglycemia would have a 66% risk of having diabetes in their remaining lifetime, and this risk increased to 88% in the presence of prediabetes. Our results are comparable to estimates from a study of Indian metropolitans reporting an expected remaining lifetime risk of diabetes of 56% for men and 65% for women from age 20 years [11].

Population differences in the propensity of diabetes as a result of differences in underlying genetic predisposition and in exposure to environmental factors will affect expected remaining lifetime risks of diabetes. The East Asian diabetes phenotype is characterized by poorer pancreatic beta-cell reserve leading to earlier glycemic decompensation and younger age of diabetes onset [32]. Higher incidence rates of type 2 diabetes have been reported in youth and adults in East Asia than in the USA and countries in Europe [13–20]. Notably, low population mortality will increase the expected remaining lifetime risk of diabetes as people are surviving to older ages and have more time to progress. Hong Kong ranks top on global ranking of life expectancies [33]. Substituting mortality rates of the Hong Kong population with rates in the CHARLS cohort resulted in falls in expected lifetime risk estimates.

The extremely high expected lifetime risk of diabetes in young Chinese people with prediabetes is alarming, as up to 9 in 10 people would decompensate metabolically. Furthermore, on average, they would live another 33 years or 52% of their remaining life span with diabetes. To our knowledge, lifetime glycemic progression in people with prediabetes has only been examined in one other study [31]. To give further perspective, at 30-year follow-up of 540 Chinese participants with IGT previously enrolled in the Da Qing Diabetes Prevention Study, 93% (126/135) of surviving people of an average age of 47 years in the control group developed diabetes [34]. Participants of the Da Qing Diabetes Prevention Study would have a higher progression rate than people with prediabetes in the present study because we have not considered IGT in our definition of prediabetes. Diabetes is associated with multiple morbidities resulting in impaired quality of life and increased use of healthcare resources. Intensive lifestyle intervention and early initiation of glucose-lowering pharmacotherapy will delay diabetes onset in individuals at risk, but these measures are resource consuming and not widely adopted [3,4]. Population-level intervention targeting the social, environmental and behavioral determinants of diabetes through policy change and public education in addition to individual-level intervention for high-risk individuals are urgently needed to reduce the toll of diabetes burden [12].

Women had a higher remaining lifetime risk and longer life years spent with diabetes than men in Hong Kong. We have previously shown that men had higher incidence rates of type 2 diabetes than women [13]. The paradoxically greater remaining lifetime risk in women most likely reflects the longer life expectancy in Hong Kong women versus men (87 years versus 82 years in 2018) [33]. Furthermore, diabetes confers greater suffering for women as excess mortality is greater in women than in men resulting in more years of life lost from diabetes in women [35].

Two studies have reported the trends in expected lifetime risk of diabetes over calendar time. A modeling study in adults in USA reported an increasing trend in the expected lifetime risk of diabetes from the period of 1985 to 1989 to 2000 to 2010 [8]. The lifetime risk estimates for type 2 diabetes in Denmark showed an increasing trend since 1996, reached a peak around 2011 followed by a decline [10]. We also observed a lower expected lifetime risk of diabetes for Hong Kong Chinese people in 2010 to 2019 compared to the preceding decade, in line with the decline in incidence of type 2 diabetes among older adults in Hong Kong [13]. The favorable shift in diabetes risks may reflect reduction in prevalent smoking [36] and adult obesity [37] as well as a series of government-led health promotion initiatives targeting healthier lifestyles in Hong Kong in the last 2 decades [38].

We observed birth cohort effects on estimation of lifetime risk of diabetes. People aged 20 years born in recent years had greater expected lifetime risks of diabetes compared with their peers born in earlier years. The most marked increase occurred in people born between 1900 and 1940 in part attributed to decline in age-standardized death rates in the Hong Kong population [39]. In addition, younger people could be more susceptible to the diabetogenic effects of nutritional transition and sedentary lifestyle, resulting in a continuous rise in expected lifetime risks of diabetes among recent generations up until 1980.

In our primary analyses, we used Markov chain model and Monte Carlo simulation to estimate remaining lifetime risk of diabetes and life years living with diabetes. The prediction performance of simulated cohort was deemed satisfactory based on the following: First, the short-term risk of diabetes in the simulated sample was similar to the observed risks in the HKDSD. Second, the estimated remaining lifetime risks were close to results derived using the modified Kaplan–Meier method, an alternative approach developed by the Framingham research group in which age was considered as the time scale and competing risk of death was adjusted [27]. Third, the estimates of years living with and without diabetes were similar to the results obtained using the Sullivan life table method [28,29]. Fourth, in sensitivity analyses in which prediabetes was defined using either FPG or HbA1c, there was minimal deviation in estimations indicating that prediabetes definition had little influence on our estimates. Fifth, exclusion of people with less than 5 years of follow-up generated similar results to the main analysis hence our estimations were minimally affected by follow-up length.

The main strength of this study is the use of a territory-wide database, which enables us to provide regionally representative estimates. In addition, the long surveillance period made it possible to compare the estimated cumulative risk with observed risk over a period of 19 years for assessing the accuracy of the estimates. There are also some limitations to note. First, the HKDSD was curated from electronic medical records of the Hong Kong HA comprising individuals who have ever had glycemic measurements on 1 occasion or more during the surveillance time. Some people were included from routine screening and others from opportunistic testing. The overall sample of the HKDSD is therefore a more selected group and potentially not entirely representative of the Hong Kong general population. To quantify the extent to which selection bias may affect our estimations, we undertook replication analysis using an independent sample of general residents in China. Lifetime risk estimates for people aged 45 years were higher in the HKDSD but the difference narrowed to 0.1% to 3.0% after substituting mortality rates in the HKDSD with rates in the CHARLS cohort. Thus, this replication analysis provides some reassurance regarding the extent to which selection bias may have affected estimates. Second, the diagnosis of diabetes was based on physician identification or glycemia measurements above diagnostic thresholds. In Hong Kong HA, screening practice was not standardized and varied over time. This could result in underestimation of undiagnosed diabetes incidence, especially in people with normoglycemia in whom diabetes screening might be less frequent because their baseline measurements were normal. Third, the HKDSD only included people who attended public hospital or clinics governed by Hong Kong HA, and people who only attended private health services were not included. However, the proportion of health services provided by private sectors to the whole health services in Hong Kong is only about 10% [21]. People might also be lost to follow if they have migrated out of Hong Kong or died outside of Hong Kong, as these events would not have been captured by the HA. According to the Hong Kong Census and Statistic Department, the net movement rate in Hong Kong from 2001 to 2019 ranged between 0.04% and 0.69% [40]. Incomplete capture of incident diabetes or death due to migration should therefore be minimal. Fourth, although it is known that prediabetes could regress to normoglycemia [41], we assumed no reversion from prediabetes to normoglycemia in our primary analyses due to potential interval censors from incomplete capture of people who achieved reversion in the HKDSD. Fifth, diabetes subtypes were not differentiated, although over 99% of Chinese people have type 2 diabetes. Exclusion of people who developed diabetes below age 20 years who were more likely to have type 1 diabetes had little effect on remaining lifetime risk estimates. Sixth, as only 1.4% of people in the HKDSD had records of 2-hour OGTT, the majority of whom were women, we did not examine the lifetime risk of diabetes in people with IGT. Given the stronger association between IGT (versus impaired fasting glucose or elevated HbA1c) and incident diabetes, we expect an even higher lifetime risk if IGT could be included [42]. Last, remaining lifetime risk for an individual is also determined by the presence of other risk factors such as low socioeconomic status, obesity, unhealthy lifestyle, and family history of diabetes. As data on these risk variables were not available for the majority of people in the HKDSD, we could not examine their effects on remaining lifetime risk.

In conclusion, the expected remaining lifetime risk of diabetes in Chinese people is high, especially in people with prediabetes. Given the known burden of cardiovascular and kidney diseases associated with diabetes, our findings call for collective action of policy makers, the community, and the individual to prevent progression to disease.

## Supporting information

S1 Text Transition probabilities estimation. (DOCX)

S2 Text The modified Kaplan–Meier method. (DOCX)

S3 Text The Sullivan life table method. (DOCX)

S1 Fig Changes in proportions of different glycemic assessments in the first 5 tests for each individual. (TIF)

S2 Fig Proportions of people with glycemic measurements to the midyear population of Hong Kong in each calendar year. (TIF)

S3 Fig Possible transitions between normoglycemia, prediabetes, diabetes, and death in Markov Chain model. (TIF)

S4 Fig Transition probabilities from normoglycemia, prediabetes, and diabetes to death when reach to the next age in HKDSD and CHARLS. CHARLS, China Health and Retirement Longitudinal Survey; HKDSD, Hong Kong Diabetes Surveillance Database.
(TIF)

S5 Fig Short-term cumulative risk of progression from normoglycemia and prediabetes to diabetes stratified by baseline age groups among all people in HKDSD. **(A)** Unadjusted for competing risk of death. **(B)** Adjusted for competing risk of death. HKDSD, Hong Kong Diabetes Surveillance Database.
(TIF)

S6 Fig Short-term cumulative risk of progression from normoglycemia and prediabetes to diabetes stratified by baseline age groups among men in in HKDSD. **(A)** Unadjusted for competing risk of death. **(B)** Adjusted for competing risk of death. HKDSD, Hong Kong Diabetes Surveillance Database.
(TIF)

S7 Fig Short-term cumulative risk of progression from normoglycemia and prediabetes to diabetes stratified by baseline age groups among women in in HKDSD. **(A)** Unadjusted for competing risk of death. **(B)** Adjusted for competing risk of death. HKDSD, Hong Kong Diabetes Surveillance Database.
(TIF)

S8 Fig Transition probabilities from normoglycemia and prediabetes to other states when reach to the next age. **(A)** People with normoglycemia. **(B)** People with prediabetes.
(TIF)

S9 Fig Expected remaining lifetime risk of progression to diabetes across index age in the period of 2001 to 2009 and 2010 to 2019 by glycemic status. (TIF)

S10 Fig Expected remaining lifetime risk of progression to diabetes across year of birth cohort stratified by index age and glycemic status. (TIF)

S11 Fig Comparison between observed and expected short-term risks stratified by onset age of prediabetes. (TIF)

S12 Fig Expected remaining lifetime risk of progression to diabetes across index age in men and women by glycemic status (simulations from age 20 years onward). (TIF)

S13 Fig Expected life years with and without diabetes after index age in men and women by glycemia status (simulations from age 20 years onward). **(A)** People with normoglycemia. **(B)** People with prediabetes.
(TIF)

S14 Fig Expected lifetime risk of progression to diabetes across index age in men and women by glycemic status (prediabetes was defined by FPG only). FPG, fasting plasma glucose.
(TIF)

S15 Fig Expected life years with and without diabetes after index age in men and women by glycemia status (prediabetes was defined by FPG only). **(A)** People with normoglycemia. **(B)** people with prediabetes. FPG, fasting plasma glucose.
(TIF)

S16 Fig Expected lifetime risk of progression to diabetes across index age in men and women by glycemic status (prediabetes was defined by HbA1c only). HbA1c, glycated hemoglobin.
(TIF)

S17 Fig Expected life years with and without diabetes after index age in men and women by glycemia status (prediabetes was defined by HbA1c only). **(A)** People with normoglycemia. **(B)** People with prediabetes. HbA1c, glycated hemoglobin.
(TIF)

S18 Fig Expected lifetime risk of progression to diabetes estimated by the modified Kaplan–Meier method across index age in men and women by glycemic status. (TIF)

S19 Fig Expected life years with and without diabetes after index age in men and women by glycemia status using the Sullivan life table method. **(A)** People with normoglycemia. **(B)** People with prediabetes.
(TIF)

S20 Fig Expected lifetime risk of progression to diabetes restricting to people with 5 years or longer follow-up across index age in men and women by glycemic status. (TIF)

S21 Fig Comparison between HKDSD and CHARLS in remaining lifetime risk of diabetes before 90 years old for people with normoglycemia or prediabetes at age 45 years. CHARLS, China Health and Retirement Longitudinal Survey; HKDSD, Hong Kong Diabetes Surveillance Database.
(TIF)

S22 Fig Comparison between HKDSD and CHARLS in remaining lifetime risk of diabetes before 90 years old for people with normoglycemia or prediabetes at age 45 years, when using the same mortality rates. CHARLS, China Health and Retirement Longitudinal Survey; HKDSD, Hong Kong Diabetes Surveillance Database.
(TIF)

S1 Table Characteristics of people included in this study from the HKDSD (2001 to 2019, *N* = 2,608,973). HKDSD, Hong Kong Diabetes Surveillance Database.
(DOCX)

S2 Table Clinical characteristics of people included in this study from the HKDSD at baseline. HKDSD, Hong Kong Diabetes Surveillance Database.
(DOCX)

S3 Table Characteristics of simulated cohort (*N* = 3,000,000). (DOCX)

S4 Table Expected remaining life years with diabetes for people with prediabetes and normoglycemia at age 20, 40, and 60 years estimated in primary analyses and sensitivity analyses. (DOCX)

S5 Table Expected remaining life years without diabetes for people with prediabetes and normoglycemia at age 20, 40, and 60 years estimated in primary analyses and sensitivity analyses. (DOCX)

S6 Table Proportions (%) of remaining life years spent with diabetes for people with prediabetes and normoglycemia at age 20, 40, and 60 years estimated in primary analyses and sensitivity analyses. (DOCX)

S7 Table Expected remaining lifetime risks of progression from prediabetes and normoglycemia to diabetes, expressed as percentages, at age 45 years estimated in CHARLS sample, HKDSD sample, and HKDSD sample with mortality rates substituted. CHARLS, China Health and Retirement Longitudinal Survey; HKDSD, Hong Kong Diabetes Surveillance Database.
(DOCX)

## Abbreviations

ADA: American Diabetes Association
CHARLS: China Health and Retirement Longitudinal Survey
FPG: fasting plasma glucose
HA: Hospital Authority
HbA1c: glycated hemoglobin
HKDSD: Hong Kong Diabetes Surveillance Database
ICD-9: International Classification of Diseases-Ninth Revision
IGT: impaired glucose tolerance
OGTT: oral glucose tolerance test

## References

[1] Tabák AG, Herder C, Rathmann W, Brunner EJ, Kivimäki M. Prediabetes: a high-risk state for diabetes development. Lancet. 2012;379(9833):2279–90. Epub 2012/06/12. doi: 10.1016/S0140-6736(12)60283-9 ; PubMed Central PMCID: PMC3891203. 22683128 PMC3891203

[2] Saeedi P, Petersohn I, Salpea P, Malanda B, Karuranga S, Unwin N, et al. Global and regional diabetes prevalence estimates for 2019 and projections for 2030 and 2045: Results from the International Diabetes Federation Diabetes Atlas, 9th edition. Diabetes Research and Clinical Practice. 2019;157:107843. doi: 10.1016/j.diabres.2019.107843 31518657

[3] Diabetes Prevention Program Research Group. Reduction in the incidence of type 2 diabetes with lifestyle intervention or metformin. New England journal of medicine. 2002;346(6):393–403. doi: 10.1056/NEJMoa012512 11832527 PMC1370926

[4] Pan X-R, Li G-w, Hu Y-H, Wang J-X, Yang W-Y, An Z-X, et al. Effects of diet and exercise in preventing NIDDM in people with impaired glucose tolerance: the Da Qing IGT and Diabetes Study. Diabetes care. 1997;20(4):537–544. doi: 10.2337/diacare.20.4.537 9096977

[5] Seshadri S, Wolf PA. Lifetime risk of stroke and dementia: current concepts, and estimates from the Framingham Study. Lancet Neurol. 2007;6(12):1106–14. Epub 2007/11/23. doi: 10.1016/S1474-4422(07)70291-0 . 18031707

[6] Narayan KMV, Boyle JP, Thompson TJ, Sorensen SW, Williamson DF. Lifetime Risk for Diabetes Mellitus in the United States. JAMA. 2003;290(14):1884–1890. doi: 10.1001/jama.290.14.1884 14532317

[7] Narayan KM, Boyle JP, Thompson TJ, Gregg EW, Williamson DF. Effect of BMI on lifetime risk for diabetes in the U.S. Diabetes Care. 2007;30(6):1562–6. Epub 2007/03/21. doi: 10.2337/dc06-2544 . 17372155

[8] Gregg EW, Zhuo X, Cheng YJ, Albright AL, Narayan KM, Thompson TJ. Trends in lifetime risk and years of life lost due to diabetes in the USA, 1985–2011: a modelling study. Lancet Diabetes Endocrinol. 2014;2(11):867–74. Epub 2014/08/17. doi: 10.1016/S2213-8587(14)70161-5 . 25128274

[9] Magliano DJ, Shaw J, Shortreed SM, Nusselder WJ, Liew D, Barr EL, et al. Lifetime risk and projected population prevalence of diabetes. Diabetologia. 2008;51(12):2179–2186. doi: 10.1007/s00125-008-1150-5 18810385

[10] Carstensen B, Rønn PF, Jørgensen ME. Lifetime risk and years lost to type 1 and type 2 diabetes in Denmark, 1996–2016. BMJ Open Diabetes Research and Care. 2021;9(1):e001065. doi: 10.1136/bmjdrc-2019-001065 33653710 PMC7929801

[11] Luhar S, Kondal D, Jones R, Anjana RM, Patel SA, Kinra S, et al. Lifetime risk of diabetes in metropolitan cities in India. Diabetologia. 2021;64(3):521–9. Epub 2020/11/24. doi: 10.1007/s00125-020-05330-1 ; PubMed Central PMCID: PMC7864818. 33225415 PMC7864818

[12] Chan JC, Lim L-L, Wareham NJ, Shaw JE, Orchard TJ, Zhang P, et al. The Lancet Commission on diabetes: using data to transform diabetes care and patient lives. The Lancet. 2020;396(10267):2019–2082. doi: 10.1016/S0140-6736(20)32374-6 33189186

[13] Luk AOY, Ke C, Lau ESH, Wu H, Goggins W, Ma RCW, et al. Secular trends in incidence of type 1 and type 2 diabetes in Hong Kong: A retrospective cohort study. PLOS Medicine. 2020;17(2):e1003052. doi: 10.1371/journal.pmed.1003052 32078650 PMC7032690

[14] Mayer-Davis EJ, Lawrence JM, Dabelea D, Divers J, Isom S, Dolan L, et al. Incidence trends of type 1 and type 2 diabetes among youths, 2002–2012. N Engl J Med. 2017;376:1419–1429. doi: 10.1056/NEJMoa1610187 28402773 PMC5592722

[15] Alangh A, Chiu M, Shah BR. Rapid increase in diabetes incidence among Chinese Canadians between 1996 and 2005. Diabetes Care. 2013;36(10):3015–3017. doi: 10.2337/dc13-0052 23723356 PMC3781544

[16] Zghebi SS, Steinke DT, Carr MJ, Rutter MK, Emsley RA, Ashcroft DM. Examining trends in type 2 diabetes incidence, prevalence and mortality in the UK between 2004 and 2014. Diabetes Obes Metab. 2017;19(11):1537–45. Epub 2017/04/08. doi: 10.1111/dom.12964 . 28387052

[17] Norhammar A, Bodegård J, Nyström T, Thuresson M, Eriksson JW, Nathanson D. Incidence, prevalence and mortality of type 2 diabetes requiring glucose-lowering treatment, and associated risks of cardiovascular complications: a nationwide study in Sweden, 2006–2013. Diabetologia. 2016;59(8):1692–701. Epub 2016/05/18. doi: 10.1007/s00125-016-3971-y . 27189067

[18] Geiss LS, Wang J, Cheng YJ, Thompson TJ, Barker L, Li Y, et al. Prevalence and incidence trends for diagnosed diabetes among adults aged 20 to 79 years, United States, 1980–2012. Jama. 2014;312(12):1218–1226. doi: 10.1001/jama.2014.11494 25247518

[19] Song SO, Lee Y-h, Kim DW, Song YD, Nam JY, Park KH, et al. Trends in diabetes incidence in the last decade based on Korean National Health Insurance claims data. Endocrinology and Metabolism. 2016;31(2):292–299. doi: 10.3803/EnM.2016.31.2.292 27302715 PMC4923414

[20] Lin C-C, Li C-I, Hsiao C-Y, Liu C-S, Yang S-Y, Lee C-C, et al. Time trend analysis of the prevalence and incidence of diagnosed type 2 diabetes among adults in Taiwan from 2000 to 2007: a population-based study. BMC Public Health. 2013;13(1):1–10. doi: 10.1186/1471-2458-13-318 23570503 PMC3626657

[21] Census and Statistics Department. Hong Kong Special Administrative Region. Thematic Household Survey Report No. 50. 2013.

[22] Wu H, Lau ESH, Yang A, Zhang X, Ma RCW, Kong APS, et al. Data Resource Profile: The Hong Kong Diabetes Surveillance Database (HKDSD). International Journal of Epidemiology. 2022;51(2):e9–e17. doi: 10.1093/ije/dyab252 34904159

[23] Census and Statistics Department. Hong Kong Special Administrative Region. Table 1A: Population by Sex and Age Group 2021 [cited 2022 March 18]. Available from: https://www.censtatd.gov.hk/en/web_table.html?id=1A#.

[24] 2. Classification and Diagnosis of Diabetes: Standards of Medical Care in Diabetes-2021. Diabetes Care. 2021;44(Suppl 1):S15–s33. Epub 2020/12/11. doi: 10.2337/dc21-S002 . 33298413

[25] de Wreede LC, Fiocco M, Putter H. The mstate package for estimation and prediction in non- and semi-parametric multi-state and competing risks models. Comput Methods Programs Biomed. 2010;99(3):261–74. Epub 2010/03/17. doi: 10.1016/j.cmpb.2010.01.001 . 20227129

[26] Evron JM, Herman WH, McEwen LN. Changes in Screening Practices for Prediabetes and Diabetes Since the Recommendation for Hemoglobin A(1c) Testing. Diabetes Care. 2019;42(4):576–84. Epub 2019/02/08. doi: 10.2337/dc17-1726 ; PubMed Central PMCID: PMC7373494. 30728220 PMC7373494

[27] Beiser A, D’Agostino RB, Sr., Seshadri S, Sullivan LM, Wolf PA. Computing estimates of incidence, including lifetime risk: Alzheimer’s disease in the Framingham Study. The Practical Incidence Estimators (PIE) macro. Stat Med. 2000;19(11–12):1495–522. Epub 2000/06/09. doi: 10.1002/(sici)1097-0258(20000615/30)19:11/12&lt;1495::aid-sim441&gt;3.0.co;2-e . 10844714

[28] Sullivan DF. A single index of mortality and morbidity. HSMHA Health Rep. 1971;86(4):347–54. Epub 1971/04/01. ; PubMed Central PMCID: PMC1937122. 5554262 PMC1937122

[29] Carol Jagger BC, Sophie Le Roy and the EHEMU team. Health Expectancy Calculation by the Sullivan Method. Third Edition[cited 2021 25 July].

[30] Zhao Y, Hu Y, Smith JP, Strauss J, Yang G. Cohort profile: the China Health and Retirement Longitudinal Study (CHARLS). Int J Epidemiol. 2014;43(1):61–8. Epub 2012/12/18. doi: 10.1093/ije/dys203 ; PubMed Central PMCID: PMC3937970. 23243115 PMC3937970

[31] Ligthart S, van Herpt TT, Leening MJ, Kavousi M, Hofman A, Stricker BH, et al. Lifetime risk of developing impaired glucose metabolism and eventual progression from prediabetes to type 2 diabetes: a prospective cohort study. Lancet Diabetes Endocrinol. 2016;4(1):44–51. Epub 2015/11/18. doi: 10.1016/S2213-8587(15)00362-9 . 26575606

[32] Chan JC, Malik V, Jia W, Kadowaki T, Yajnik CS, Yoon KH, et al. Diabetes in Asia: epidemiology, risk factors, and pathophysiology. Jama. 2009;301(20):2129–40. Epub 2009/05/28. doi: 10.1001/jama.2009.726 . 19470990

[33] The World Bank. World Bank Open Data [cited 2021 18 Nov]. Available from: https://data.worldbank.org/.

[34] Gong Q, Zhang P, Wang J, Ma J, An Y, Chen Y, et al. Morbidity and mortality after lifestyle intervention for people with impaired glucose tolerance: 30-year results of the Da Qing Diabetes Prevention Outcome Study. The Lancet Diabetes & Endocrinology. 2019;7(6):452–461. 10.1016/S2213-8587(19)30093-2 31036503 PMC8172050

[35] Zhang ZJ, Zhao G, Yu C, Bi Y, Zhang Q, Song Y. Diabetic women suffer more years of life lost than diabetic men. Int J Endocrinol. 2014;2014:208369. Epub 2014/12/30. doi: 10.1155/2014/208369 ; PubMed Central PMCID: PMC4269083. 25544840 PMC4269083

[36] Tobacco and Alcohol Control Office DoHTGotHKSAR. Prevalence of current smokers (aged 15 and over) 2012 [cited March 22 2022]. Available from: https://www.taco.gov.hk/t/english/infostation/infostation_sta_01.html.

[37] Ko GT, Tang JS, Chan JC. Worsening trend of central obesity despite stable or declining body mass index in Hong Kong Chinese between 1996 and 2005. Eur J Clin Nutr. 2010;64(5):549–52. Epub 2010/03/25. doi: 10.1038/ejcn.2010.49 . 20332802

[38] Department of Health. The Government of the Hong Kong Special Administrative Region. Promoting Health in Hong Kong: A Strategic Framework for Prevention and Control of Non-communicable Diseases 2008 [accessed 18 March 2022]. Available from: https://www.change4health.gov.hk/filemanager/common/image/strategic_framework/promoting_health/promoting_health_e.pdf.

[39] The Centre for Health Protection. Department of Health. The Hong Kong Special Administrative Region. Age-standardised Death Rate, 1981–2020 [updated 14th September 2021]. Available from: https://www.chp.gov.hk/en/statistics/data/10/27/114.html.

[40] The Census and Statistics Department of Hong Kong. Population Estimates [updated 22 April, 2021; cited 18 November, 2021]. Available from: https://www.censtatd.gov.hk/tc/scode150.html.

[41] Lazo-Porras M, Bernabe-Ortiz A, Ruiz-Alejos A, Smeeth L, Gilman RH, Checkley W, et al. Regression from prediabetes to normal glucose levels is more frequent than progression towards diabetes: The CRONICAS Cohort Study. Diabetes Res Clin Pract. 2020;163:107829. Epub 2019/08/30. doi: 10.1016/j.diabres.2019.107829 ; PubMed Central PMCID: PMC7239508. 31465811 PMC7239508

[42] Schmidt MI, Bracco PA, Yudkin JS, Bensenor IM, Griep RH, Barreto SM, et al. Intermediate hyperglycaemia to predict progression to type 2 diabetes (ELSA-Brasil): an occupational cohort study in Brazil. Lancet Diabetes Endocrinol. 2019;7(4):267–77. Epub 2019/02/26. doi: 10.1016/S2213-8587(19)30058-0 . 30803929

