# Supplementary material for: Lifetime risk of developing diabetes in Chinese people with normoglycemia or prediabetes: A modeling study
Source: PLoS Med. 2022 Jul 21;19(7):e1004045. doi: 10.1371/journal.pmed.1004045 (PMC9302798; doi:10.1371/journal.pmed.1004045)
Supplement: S1 Text — (DOCX) [file pmed.1004045.s001.docx]

**S1 Text. Transition probabilities estimation**

Denote the state of a person as X(t) and the possible states included: 1, 2, 3, …, S. The instantaneous risk of a transition from state g to state h at time t was defined as:

$$\alpha_{gh}\left( t \right)=\lim_{\Delta t\to0}\frac{P\left( X\left( t+\Delta t \right)=\left. h \right|X\left( t \right)=g \right)}{\Delta t}$$

The cumulative transition hazard (transition intensities) was defined as:

$$A_{gh}\left( t \right)=\int_{0}^{t} \alpha_{gh}\left( \mu\right)ⅆ\mu$$

If the transition from stage g to state h was impossible then $A_{gh}\left( t \right)$=0. We gathered these transition intensities into a S×S matrix $A\left( t \right)$. The diagonal elements were defined as $A_{gg}\left( t \right)={1-\Sigma}_{h\neq g}A_{gh}\left( t \right)$, indicating the probability of remaining state g was defined as:

1$-$sum of probabilities of transition from state g to other states

According to the Markov assumption, the future state depends on the past state only through the present state. Based on the matrix A(t), we calculated the transition probability matrix from time s to a future time t by means of product integral:

$$\mathbf{P}\left( s,t \right)=\prod_{\left( s,t \right]} \left( d\mathbf{A}\left( u \right) \right)$$

The matrix $\mathbf{P}\left( s,t \right)$is like:

| To  From | 1 | 2 | 3 | … | S |
| --- | --- | --- | --- | --- | --- |
| 1 | P_11_ | P_12_ | P_13_ | … | P_1S_ |
| 2 | P_21_ | P_22_ | P_23_ | … | P_2S_ |
| 3 | P_31_ | P_32_ | P_33_ | … | P_3S_ |
| … | … | … | … | … | … |
| S | P_S1_ | P_S2_ | P_S3_ | … | P_SS_ |

In our case, a person had four states: normoglycaemia, prediabetes, diabetes, and death, and there was no reversion. We calculated the $A_{gh}\left( t \right)$ using the Cox model with the glycaemic state as a time-varying event and without covariate adjusted, using the coxph() function implemented in survival package [1].

Our prime interest was the transition probability from an index age (y) to the next age, therefore the transition probability matrix in our model was given by:

$$\mathbf{P}\left( y,y+1 \right)=\mathbf{A}(y+1)$$

Using the above method, we calculated the transition probability matrix from each index age to the next age:

| To  From | Normoglycaemia | Prediabetes | Diabetes | Death |
| --- | --- | --- | --- | --- |
| Normoglycaemia | P_nn_ | P_np_ | P_nd_ | P_ns_ |
| Prediabetes | 0 | P_pp_ | P_pd_ | P_ps_ |
| Diabetes | 0 | 0 | P_dd_ | P_ds_ |
| Death | 0 | 0 | 0 | P_ss_ |

**References**

1. Terry M Therneau TL, Atkinson Elizabeth, Crowson Cynthia. Package ‘survival’2022 March 20 2022 [cited 2022 March 20 2022]. Available from: <https://cran.r-project.org/web/packages/survival/survival.pdf>.
