# Supplementary material for: Lifetime risk of developing diabetes in Chinese people with normoglycemia or prediabetes: A modeling study
Source: PLoS Med. 2022 Jul 21;19(7):e1004045. doi: 10.1371/journal.pmed.1004045 (PMC9302798; doi:10.1371/journal.pmed.1004045)
Supplement: S2 Text — (DOCX) [file pmed.1004045.s002.docx]

**S2 Text. The modified Kaplan-Meier method**

A modified Kaplan–Meier method developed by the Framingham study was applied to estimate the remaining lifetime risk of progression from pre-diabetes to diabetes for people who aged 20 years old. [1] Conventionally, Kaplan-Meier survival analysis takes the follow-up time as the time variable. In the modified method, age at entry into the study is set as the left-truncation variable and survival age (free of diabetes and alive) is used as the time variable.

Individuals who achieved a certain age j free of diabetes at some point during follow-up constituted the population at risk for age j (risk set, Rj). If an individual progressed to diabetes, died or was censored at age j, he or she was removed from the risk set for age j+1 and older. If an individual entered the study at age j+1, he or she would be counted into the risk set for age j+1. For the lifetime risk at 20 years, hazards (hj), age-specific incidences (fj), cumulative incidences (Fj), and survival probabilities (Sj) were calculated according to the standard Kaplan–Meier method for each age j (assuming F19 = 0 and S19 = 1):

hj = ej / Rj (ej is the sum of incident diabetes at age j); fj = hj × Sj–1;

$\mathrm{Fj}=\sum_{j=20}^{\mathrm{maximum} age} \mathrm{fj}$; Sj = 1–Fj.

However, Fj was the cumulative incidence that applied to individuals who survive through age j-1, which might be biased by the competing risk of death and cause overestimation of the lifetime risk. Therefore, a separate survival function (Uj) with death was included as an event alongside diabetes to adjust for the competing risk of death. The adjusted lifetime risk was calculated as follows (assuming F19 = 0 and U19 = 0):

hj = ej / Rj (ej is the sum of incident diabetes at age j);

$\mathrm{Uj}=1-\sum_{j=20}^{\mathrm{maximum} age} \left( \frac{\mathrm{cj}}{\mathrm{Rj}} \right)\times Uj-1$ (cj is the sum of incident diabetes or death at age j);

fj = hj × Uj – 1; $\mathrm{Fj}=\sum_{j=20}^{\max survival age} \mathrm{fj}$.

**References**

1. Beiser A, D'Agostino RB, Sr., Seshadri S, Sullivan LM, Wolf PA. Computing estimates of incidence, including lifetime risk: Alzheimer's disease in the Framingham Study. The Practical Incidence Estimators (PIE) macro. Stat Med. 2000;19(11-12):1495-522. Epub 2000/06/09. doi: 10.1002/(sici)1097-0258(20000615/30)19:11/12<1495::aid-sim441>3.0.co;2-e. PubMed PMID: 10844714.
