# Supplementary material for: Lifetime risk of developing diabetes in Chinese people with normoglycemia or prediabetes: A modeling study
Source: PLoS Med. 2022 Jul 21;19(7):e1004045. doi: 10.1371/journal.pmed.1004045 (PMC9302798; doi:10.1371/journal.pmed.1004045)
Supplement: S3 Text — (DOCX) [file pmed.1004045.s003.docx]

**S3 Text. The Sullivan life table method**

The Sullivan life table is a tool to estimate life years with or without a state of health condition by combining traditional life table with prevalence data [1, 2]. It requires age-specific mortality rates and age-specific prevalence of the population in healthy and unhealthy states. Based on the HKDSD (2001-2019), we separately calculated age- and sex- specific mortality rates and prevalence of diabetes at each index age for people with and without prediabetes. The life table started from the age 0 years and through to 110 years. Overall, 500 bootstrap sample data sets were generated to construct nonparametric confidence intervals of remaining life years with and without diabetes and proportions of life years spent with diabetes to the remaining life years.

**References**

1. Carol Jagger BC, Sophie Le Roy and the EHEMU team. Health Expectancy Calculation by the Sullivan Method. Third Edition[cited 2021 25 July].

2. Sullivan DF. A single index of mortality and morbidity. HSMHA Health Rep. 1971;86(4):347-54. Epub 1971/04/01. PubMed PMID: 5554262; PubMed Central PMCID: PMCPMC1937122.
