# Supplementary figures and images for: Lifetime risk of developing diabetes in Chinese people with normoglycemia or prediabetes: A modeling study
Source: PLoS Med. 2022 Jul 21;19(7):e1004045. doi: 10.1371/journal.pmed.1004045 (PMC9302798; doi:10.1371/journal.pmed.1004045)

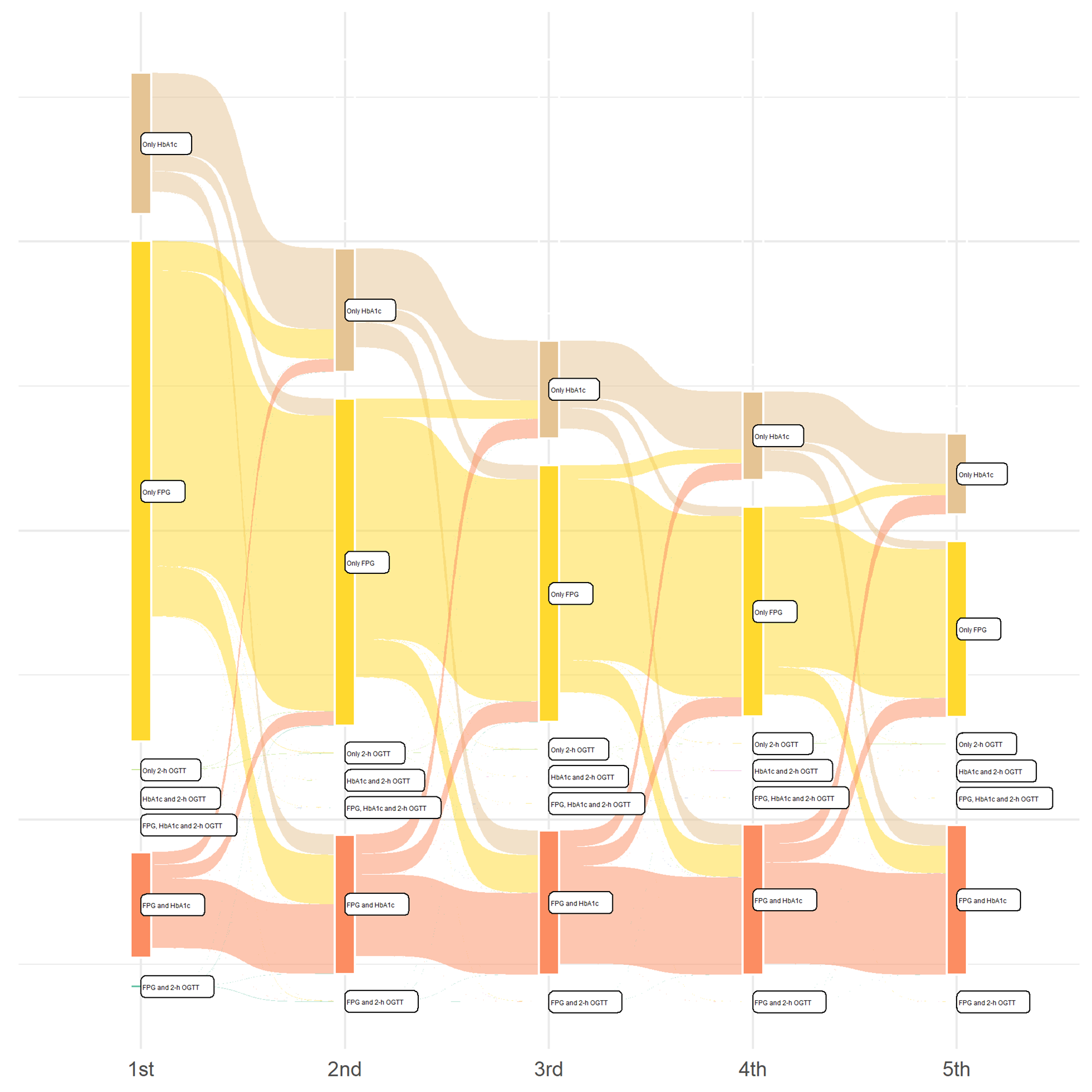

Supplement: S1 Fig — (TIF) [file pmed.1004045.s004.tif]

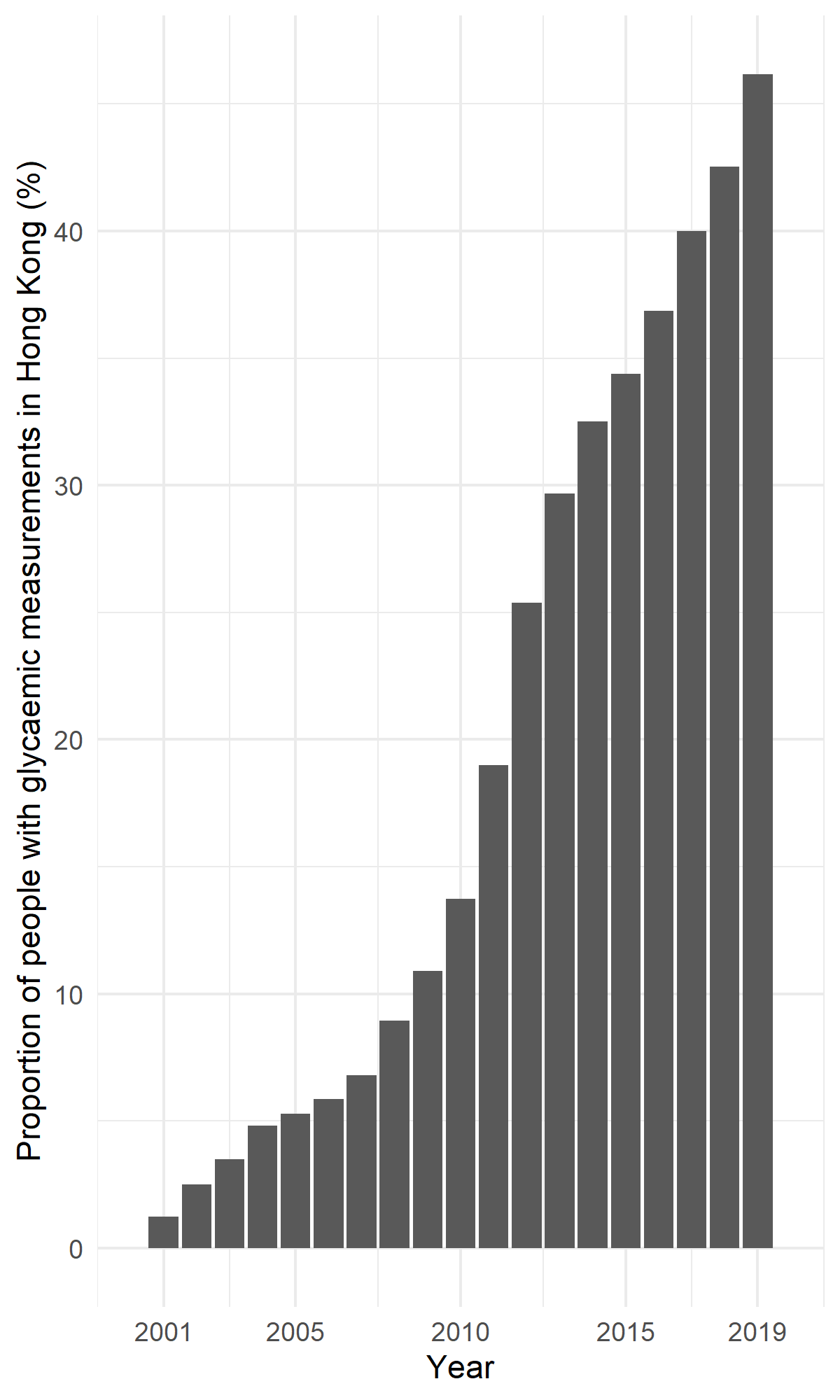

Supplement: S2 Fig — (TIF) [file pmed.1004045.s005.tif]

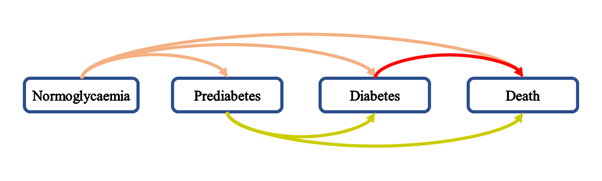

Supplement: S3 Fig — (TIF) [file pmed.1004045.s006.tif]

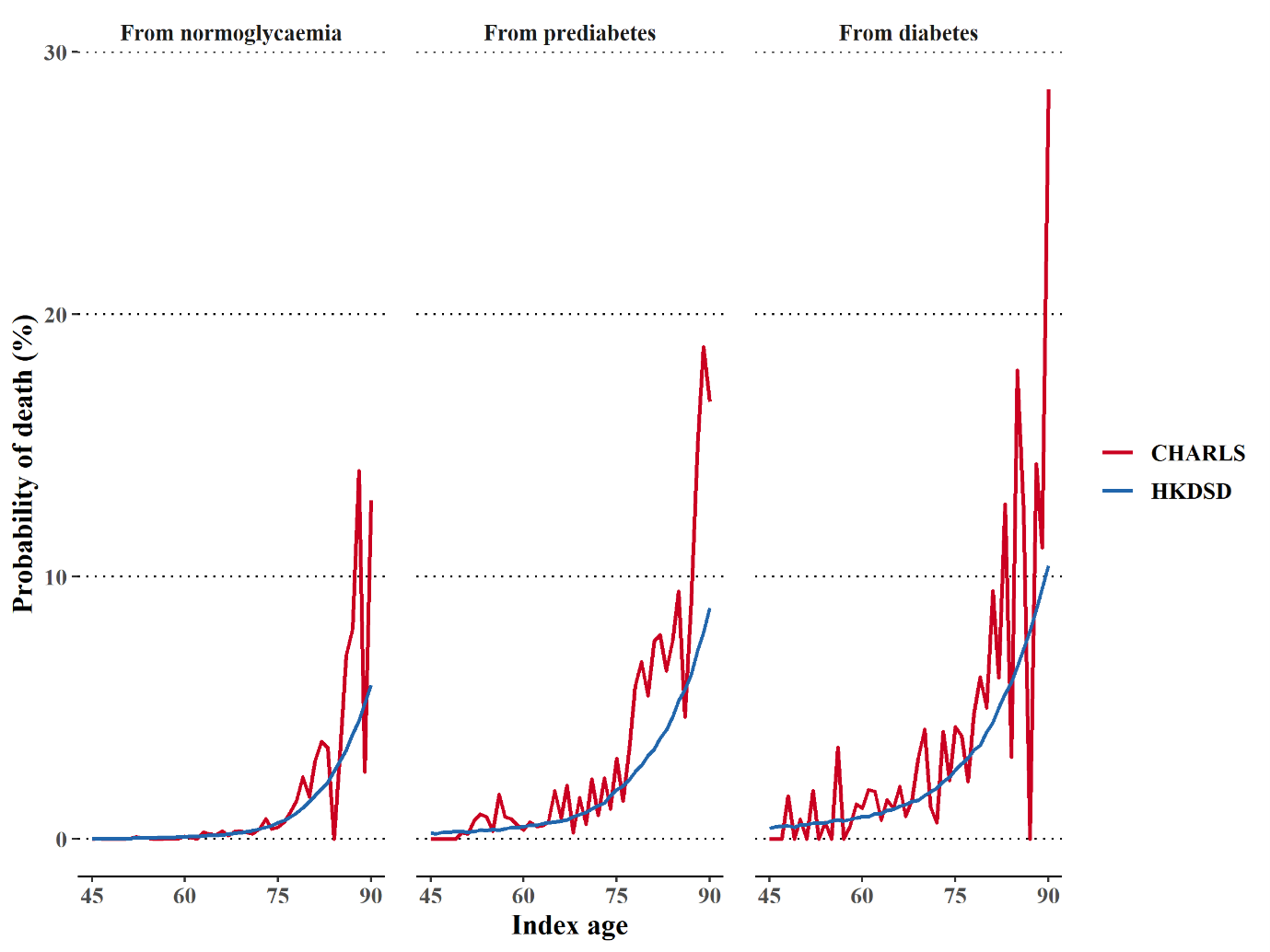

Supplement: S4 Fig — CHARLS, China Health and Retirement Longitudinal Survey; HKDSD, Hong Kong Diabetes Surveillance Database. (TIF) [file pmed.1004045.s007.tif]

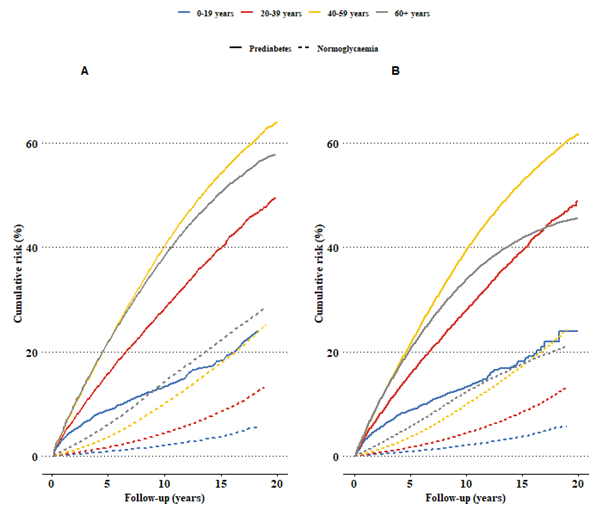

Supplement: S5 Fig — (A) Unadjusted for competing risk of death. (B) Adjusted for competing risk of death. HKDSD, Hong Kong Diabetes Surveillance Database. (TIF) [file pmed.1004045.s008.tif]

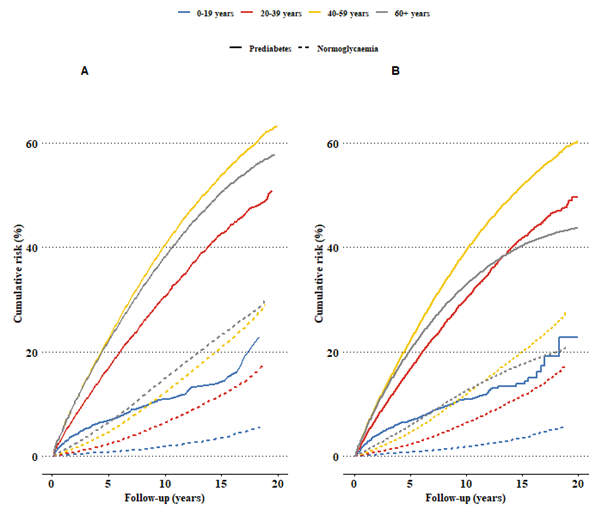

Supplement: S6 Fig — (A) Unadjusted for competing risk of death. (B) Adjusted for competing risk of death. HKDSD, Hong Kong Diabetes Surveillance Database. (TIF) [file pmed.1004045.s009.tif]

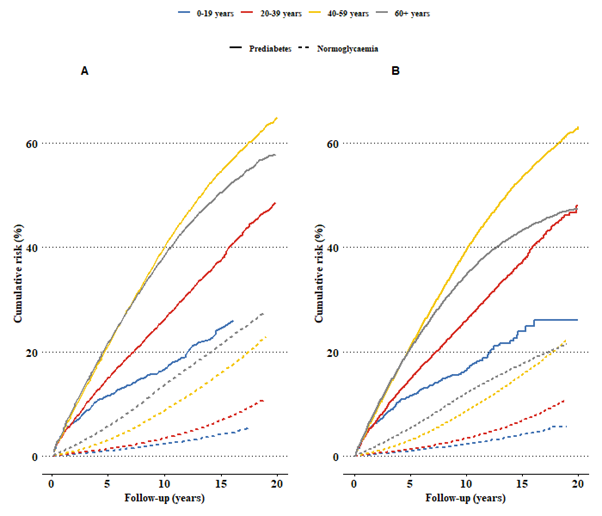

Supplement: S7 Fig — (A) Unadjusted for competing risk of death. (B) Adjusted for competing risk of death. HKDSD, Hong Kong Diabetes Surveillance Database. (TIF) [file pmed.1004045.s010.tif]

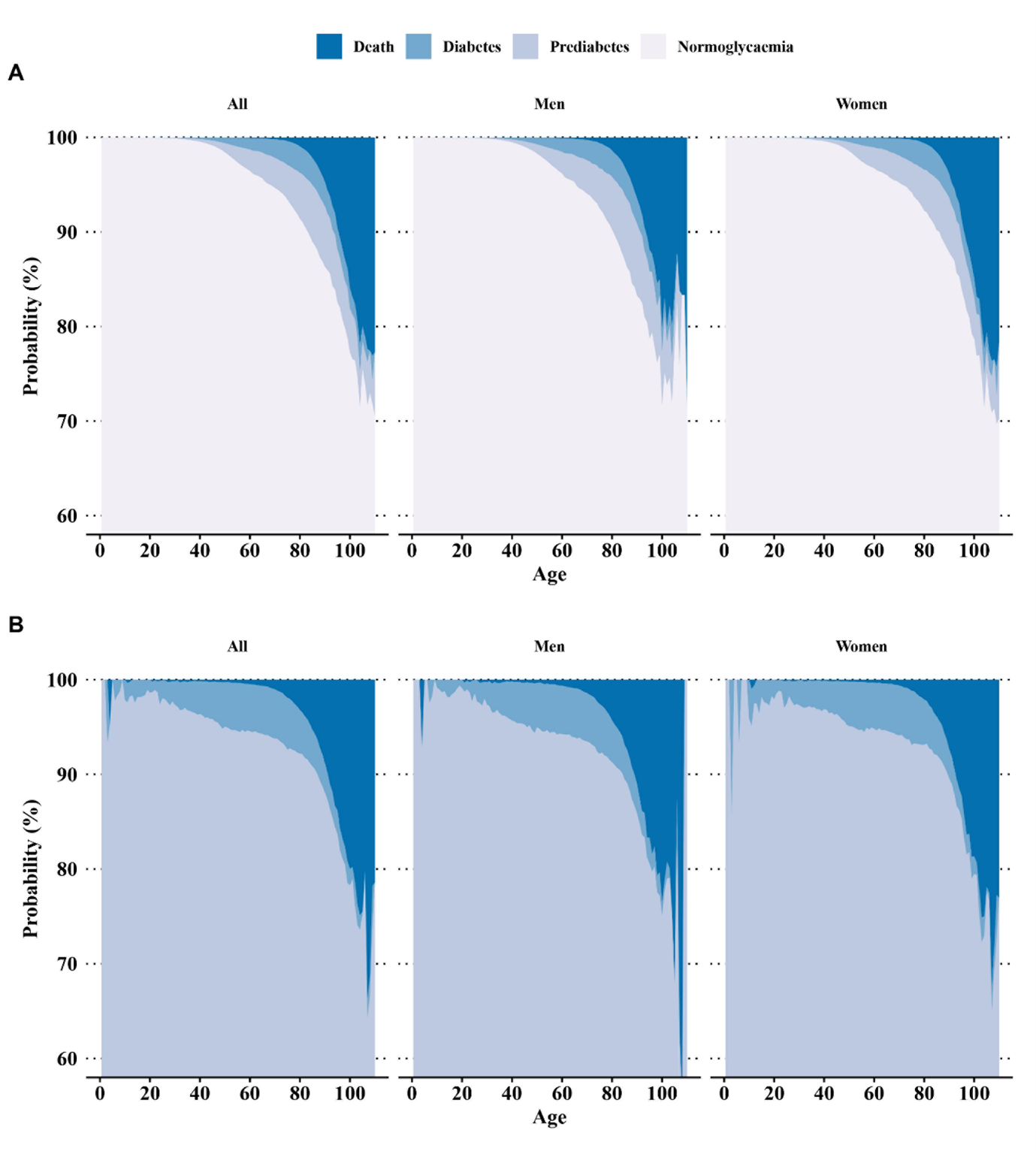

Supplement: S8 Fig — (A) People with normoglycemia. (B) People with prediabetes. (TIF) [file pmed.1004045.s011.tif]

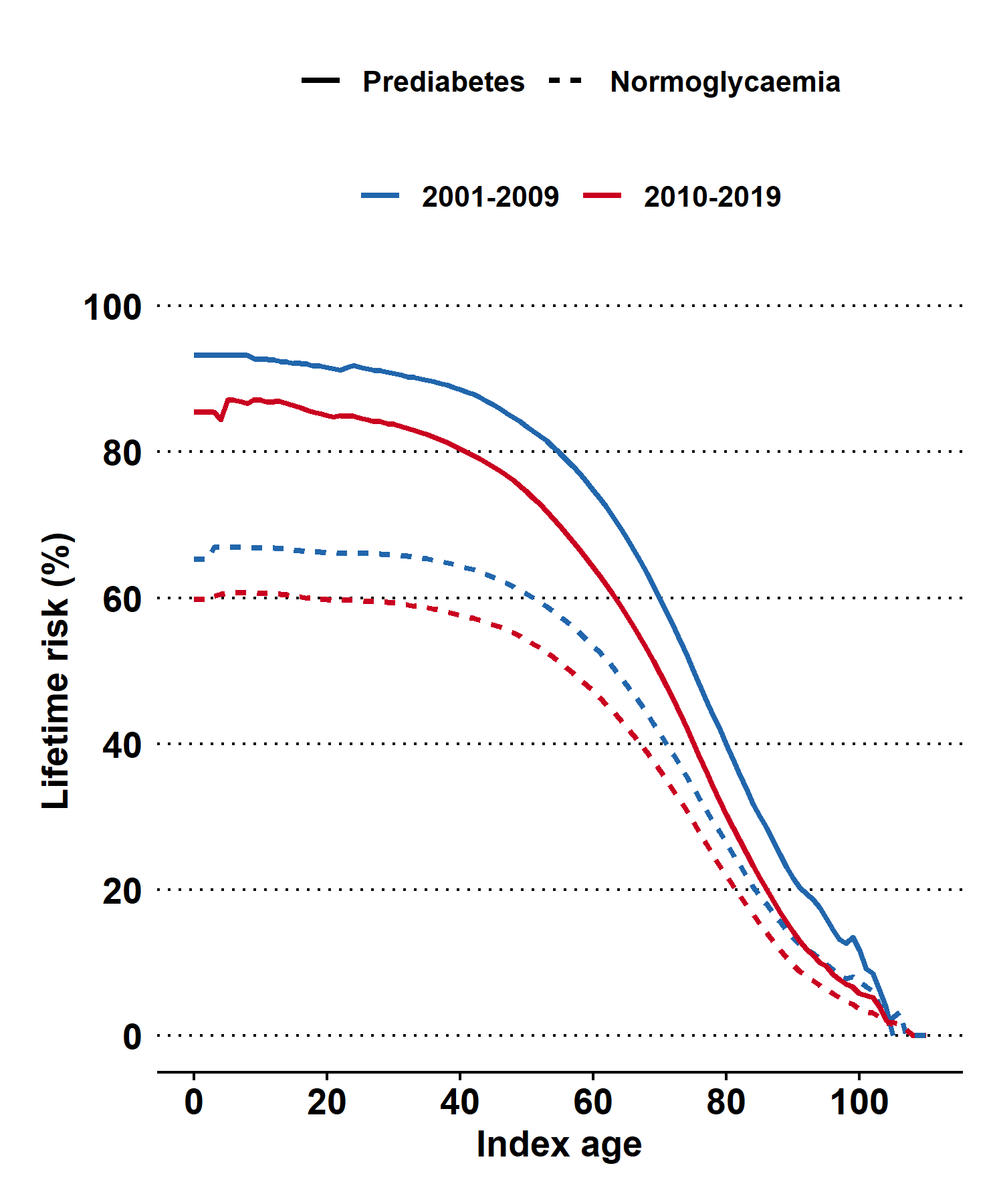

Supplement: S9 Fig — (TIF) [file pmed.1004045.s012.tif]

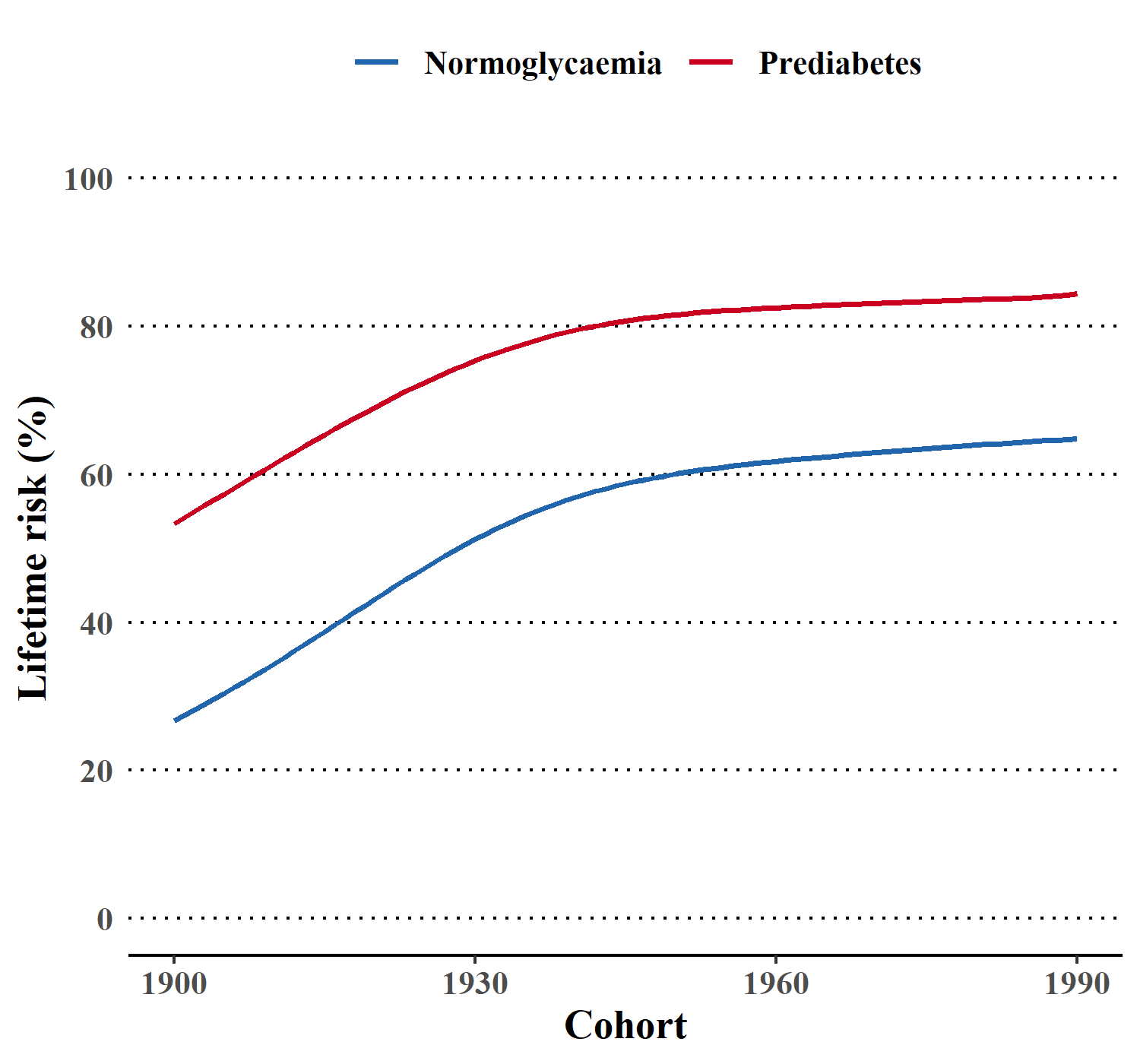

Supplement: S10 Fig — (TIF) [file pmed.1004045.s013.tif]

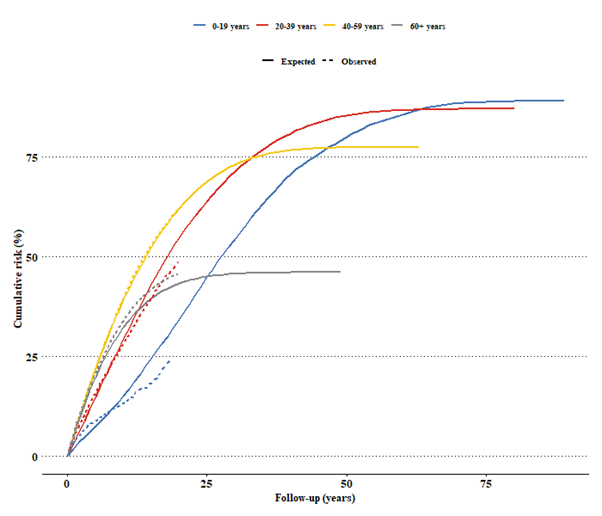

Supplement: S11 Fig — (TIF) [file pmed.1004045.s014.tif]

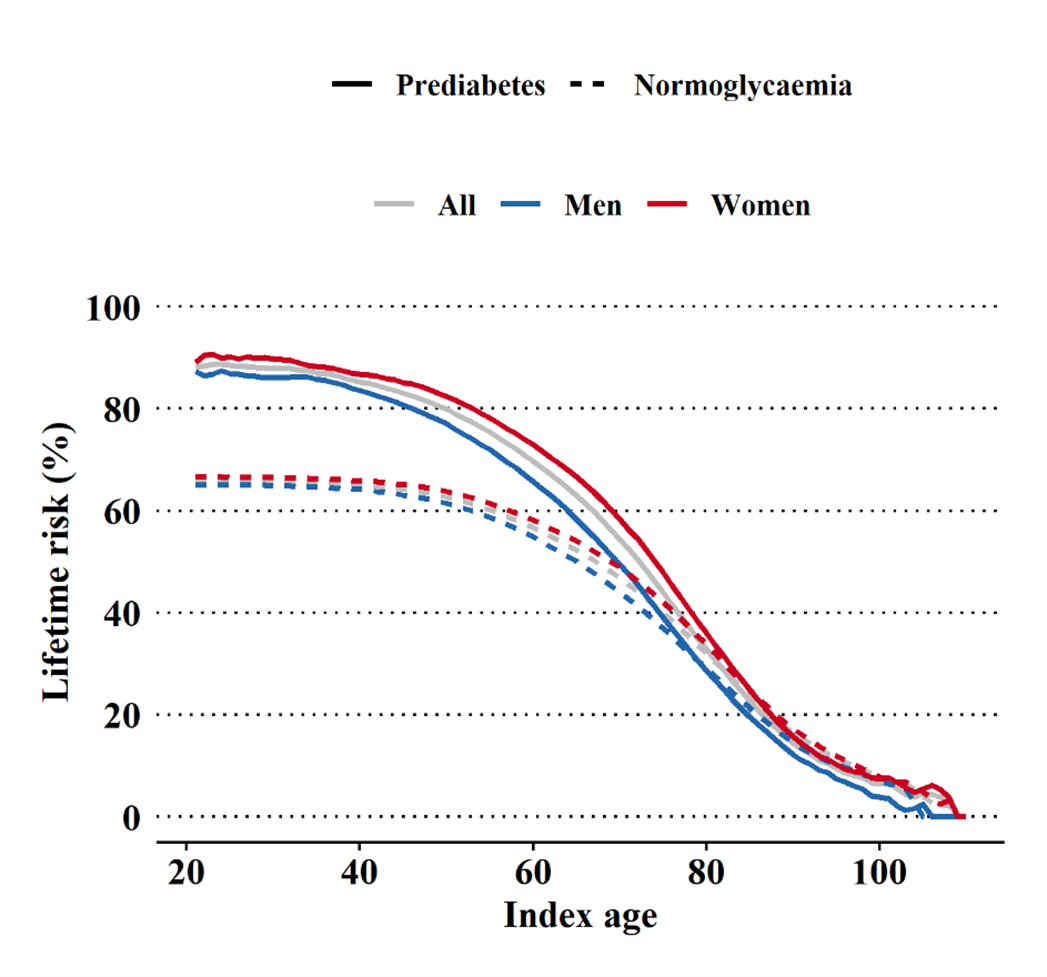

Supplement: S12 Fig — (TIF) [file pmed.1004045.s015.tif]

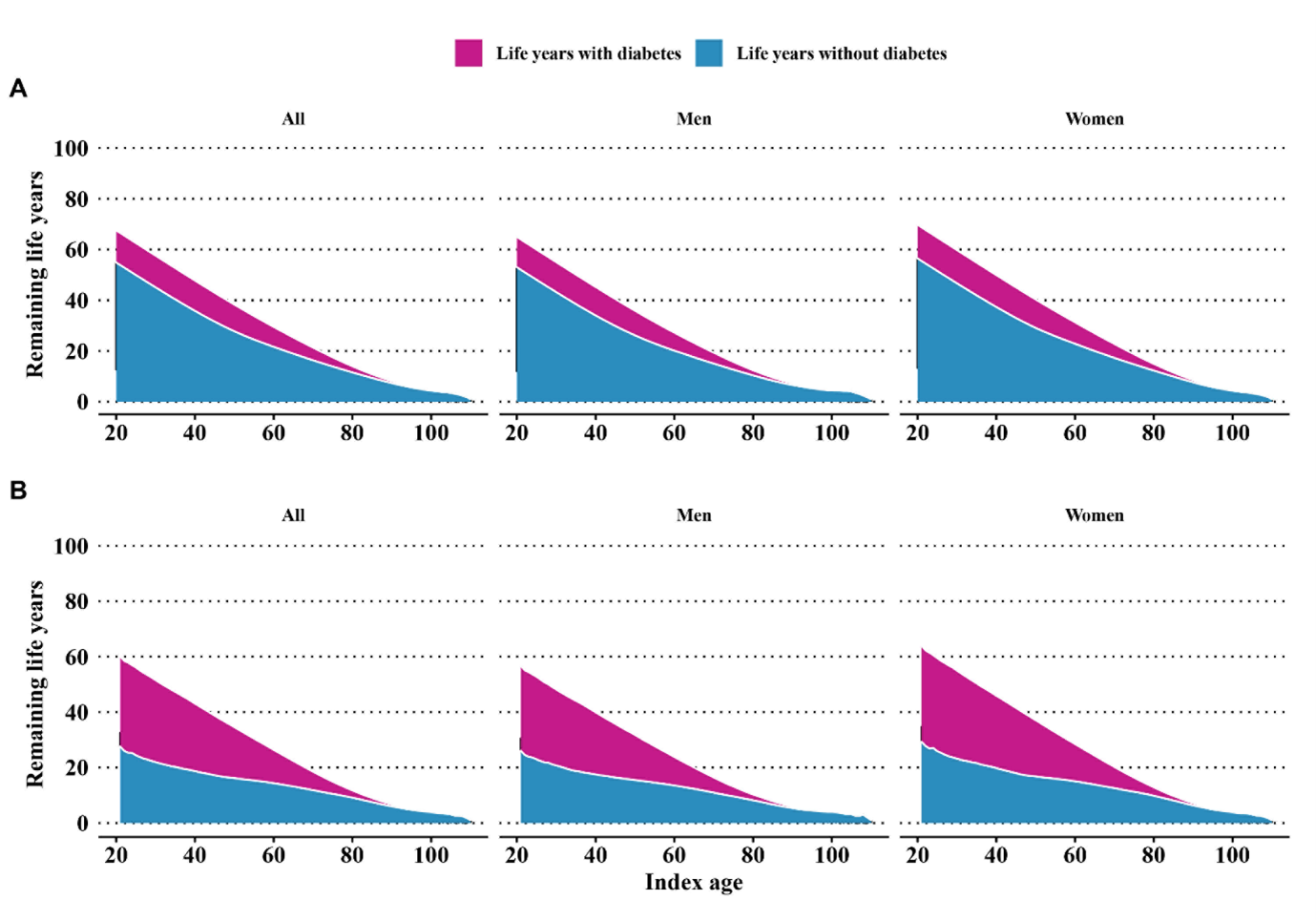

Supplement: S13 Fig — (A) People with normoglycemia. (B) People with prediabetes. (TIF) [file pmed.1004045.s016.tif]

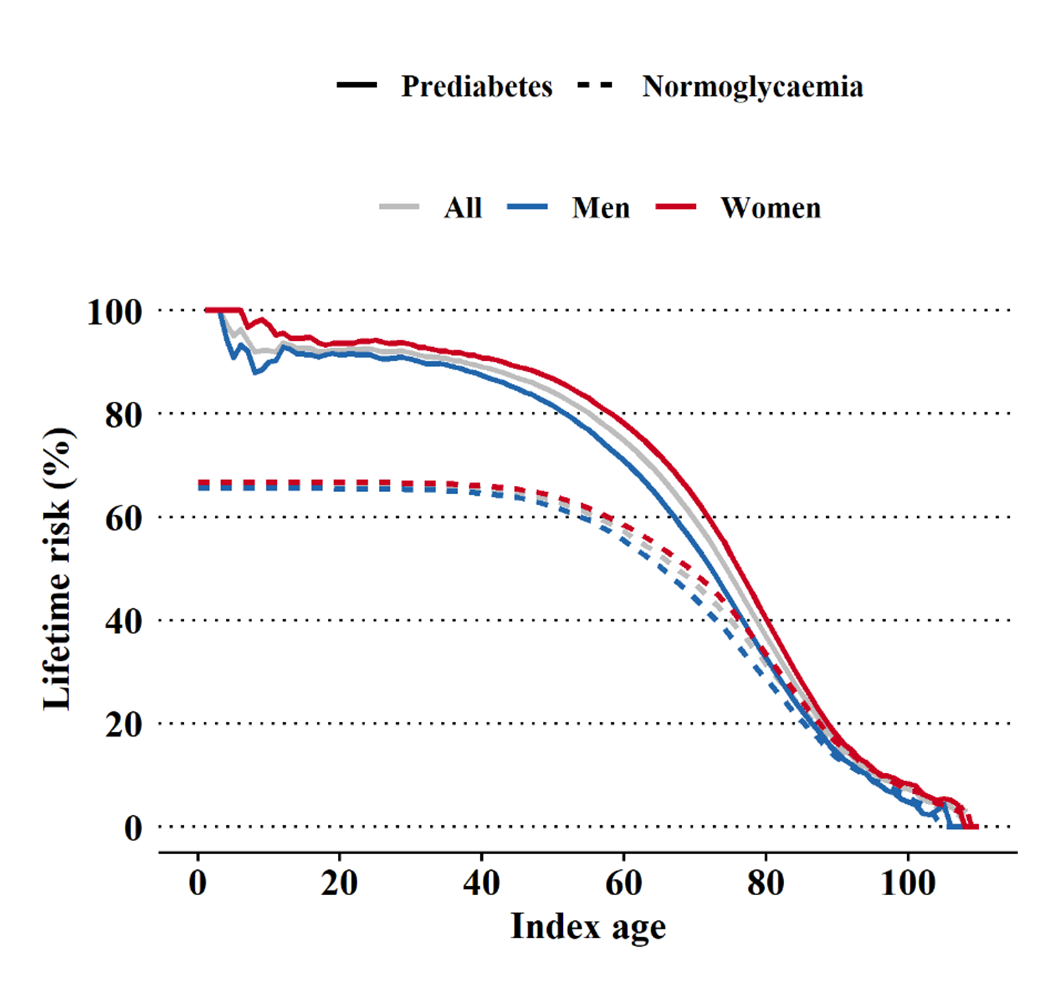

Supplement: S14 Fig — FPG, fasting plasma glucose. (TIF) [file pmed.1004045.s017.tif]

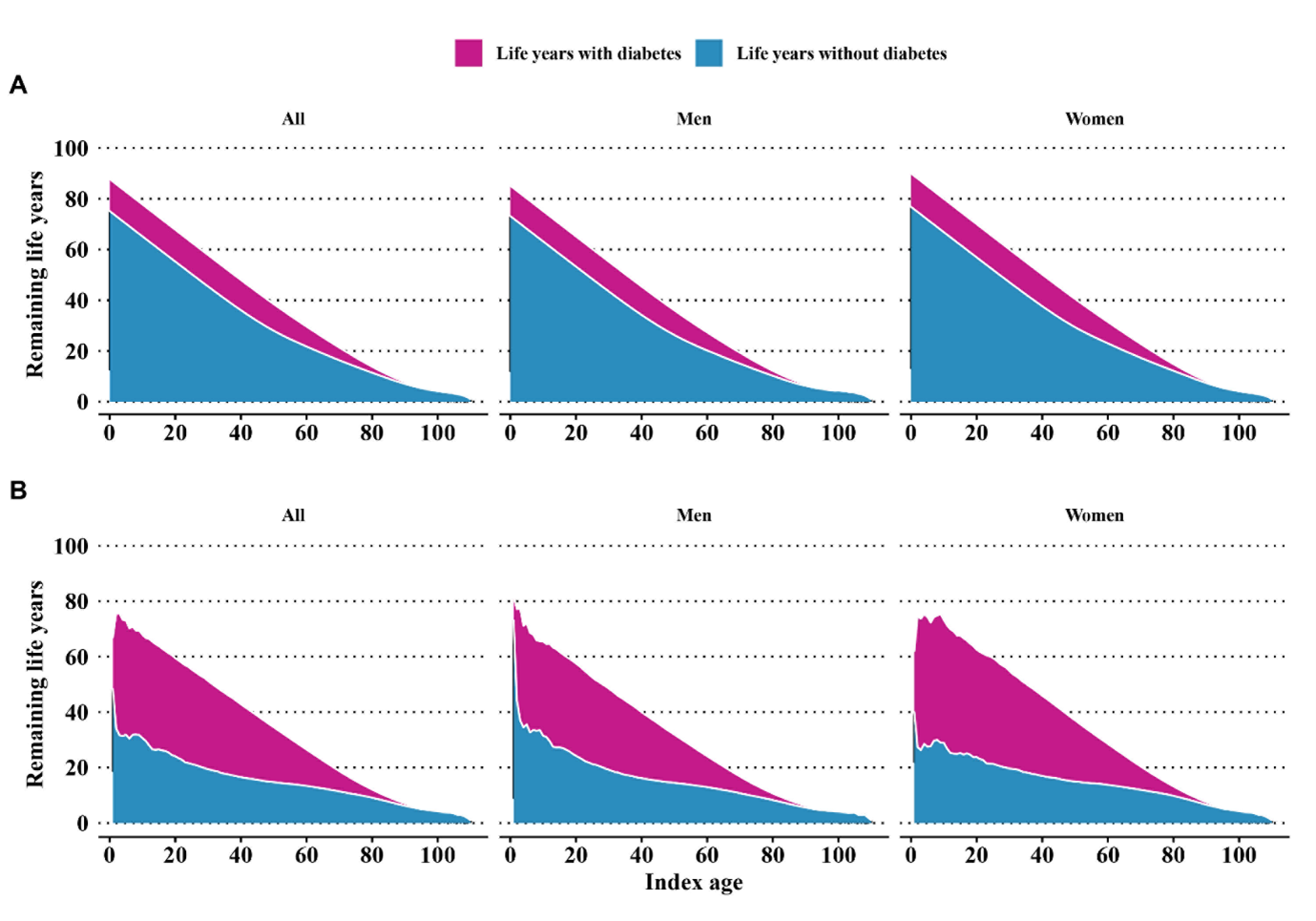

Supplement: S15 Fig — (A) People with normoglycemia. (B) people with prediabetes. FPG, fasting plasma glucose. (TIF) [file pmed.1004045.s018.tif]

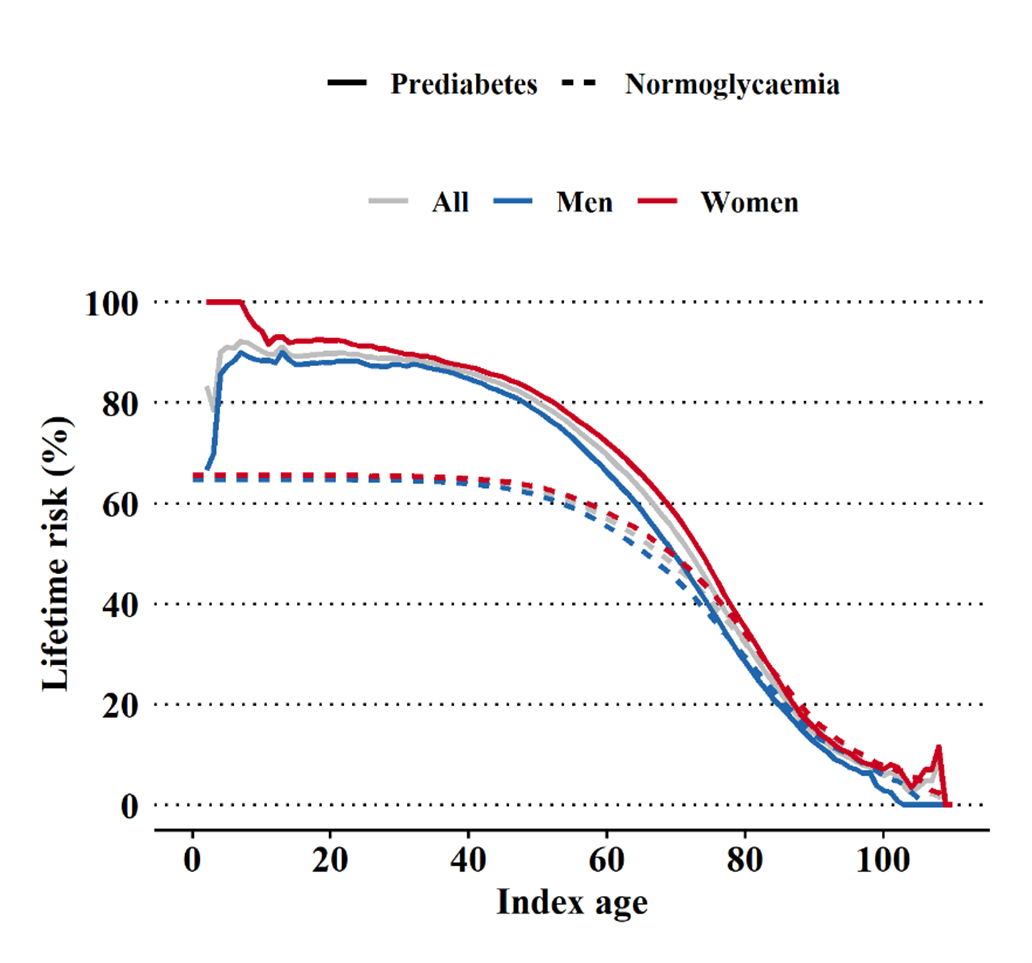

Supplement: S16 Fig — HbA1c, glycated hemoglobin. (TIF) [file pmed.1004045.s019.tif]

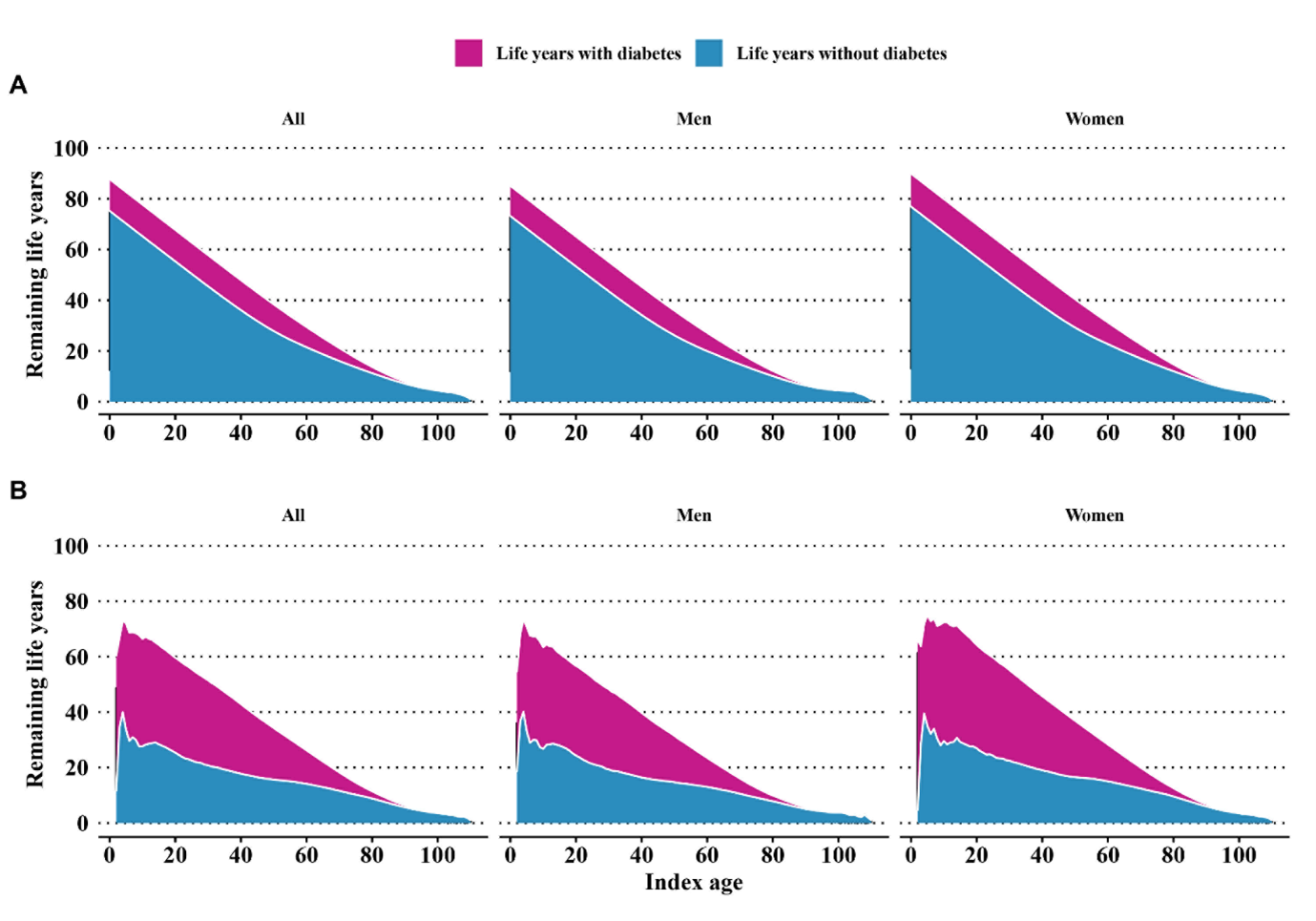

Supplement: S17 Fig — (A) People with normoglycemia. (B) People with prediabetes. HbA1c, glycated hemoglobin. (TIF) [file pmed.1004045.s020.tif]

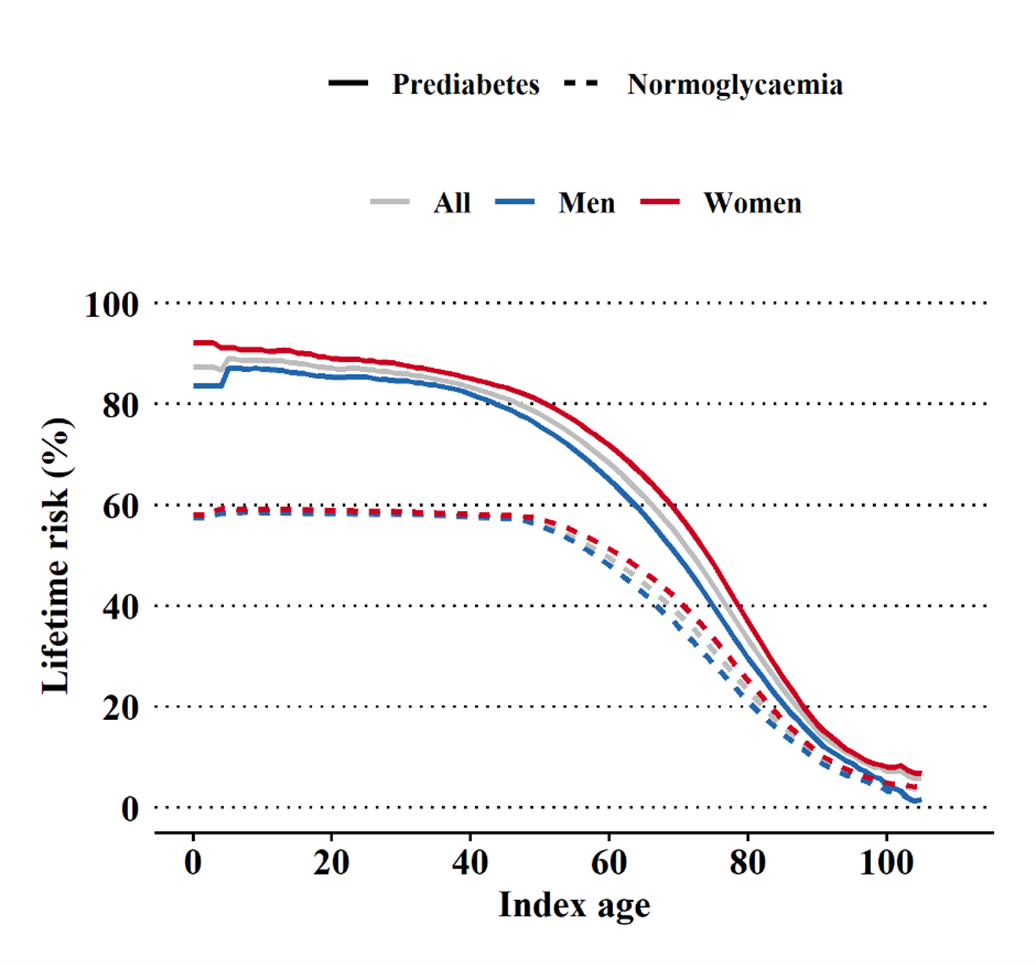

Supplement: S18 Fig — (TIF) [file pmed.1004045.s021.tif]

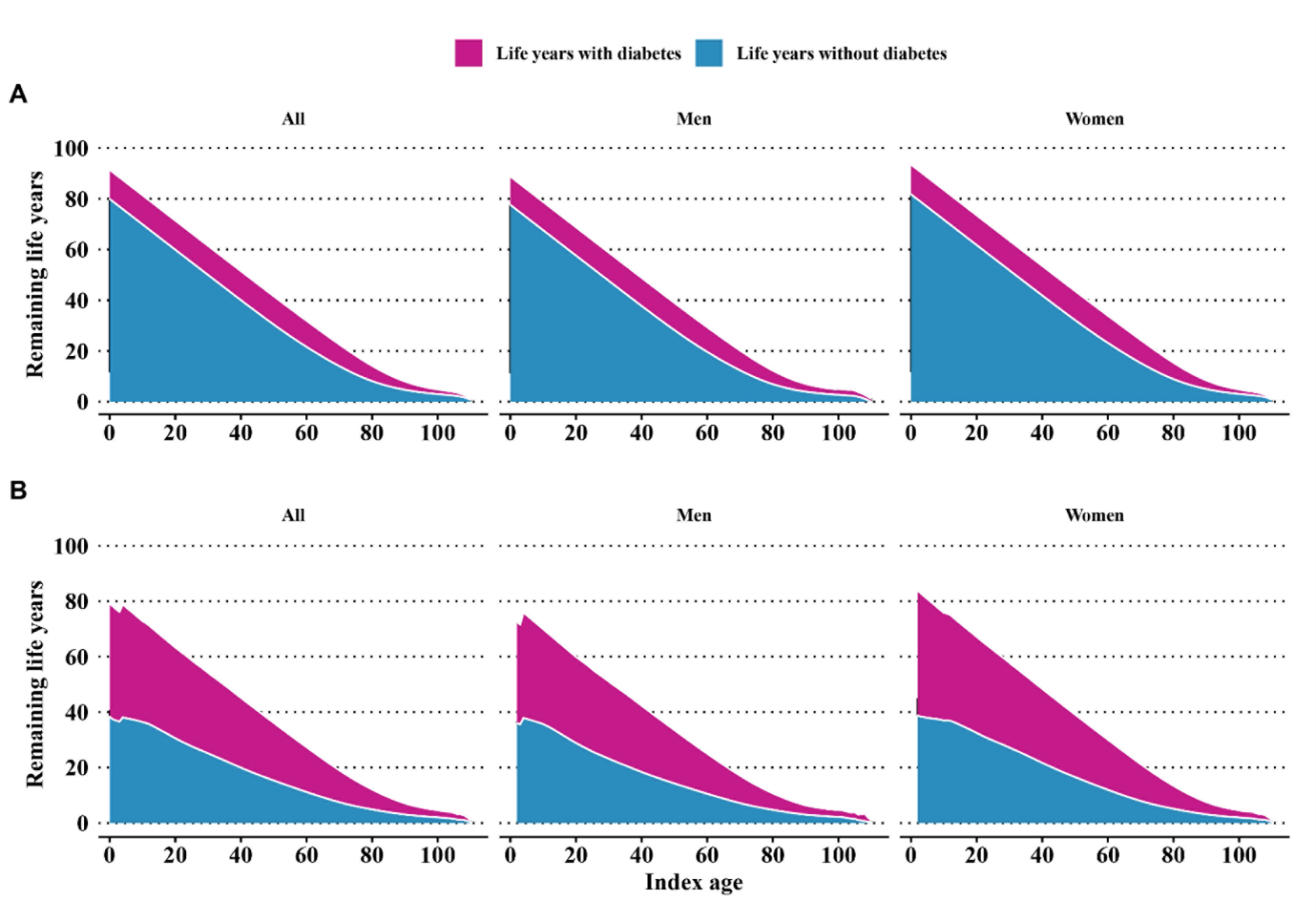

Supplement: S19 Fig — (A) People with normoglycemia. (B) People with prediabetes. (TIF) [file pmed.1004045.s022.tif]

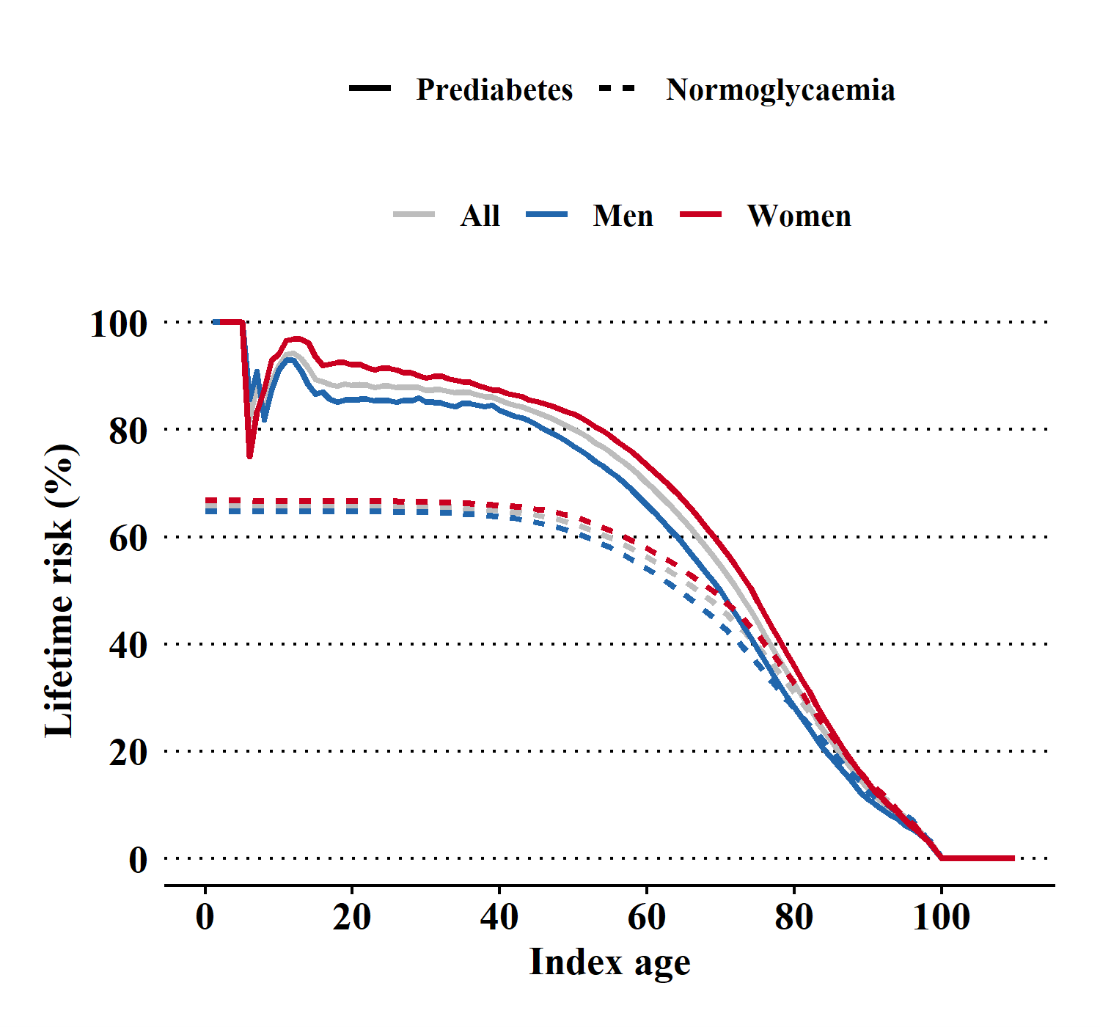

Supplement: S20 Fig — (TIF) [file pmed.1004045.s023.tif]

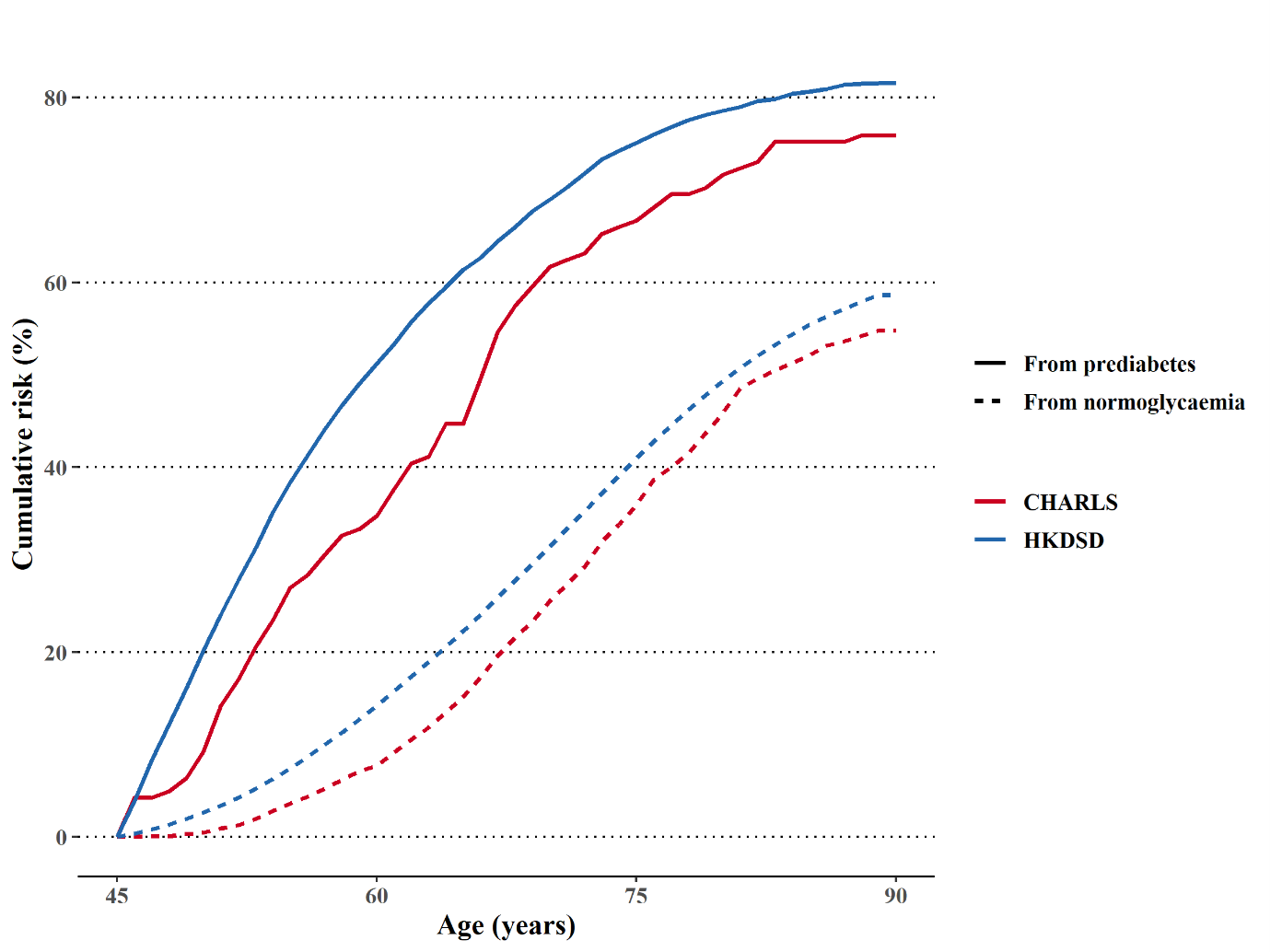

Supplement: S21 Fig — CHARLS, China Health and Retirement Longitudinal Survey; HKDSD, Hong Kong Diabetes Surveillance Database. (TIF) [file pmed.1004045.s024.tif]

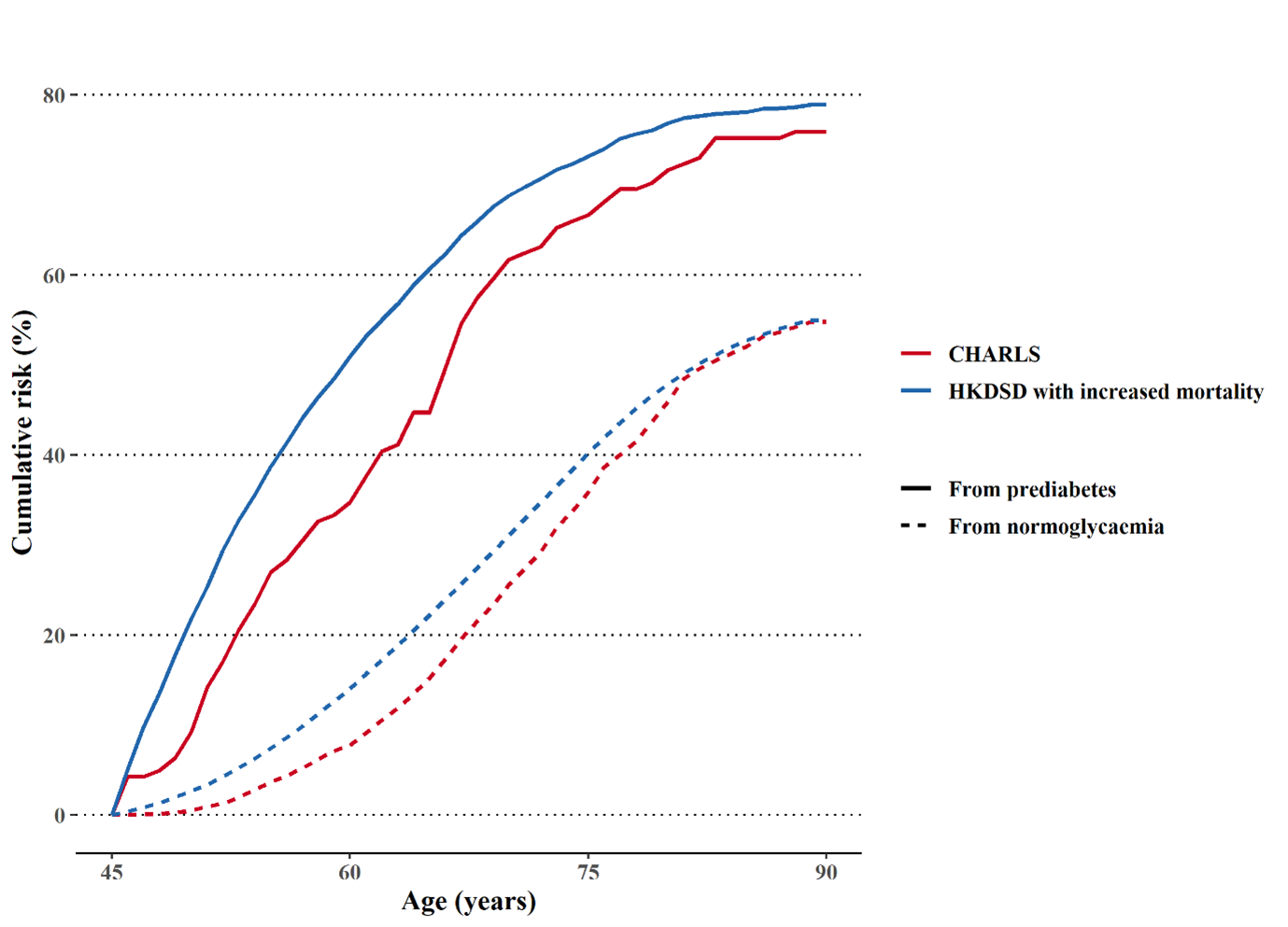

Supplement: S22 Fig — CHARLS, China Health and Retirement Longitudinal Survey; HKDSD, Hong Kong Diabetes Surveillance Database. (TIF) [file pmed.1004045.s025.tif]
