# Supplementary material for: Lifetime risk of developing diabetes in Chinese people with normoglycemia or prediabetes: A modeling study
Source: PLoS Med. 2022 Jul 21;19(7):e1004045. doi: 10.1371/journal.pmed.1004045 (PMC9302798; doi:10.1371/journal.pmed.1004045)
Supplement: S1 Table — HKDSD, Hong Kong Diabetes Surveillance Database. (DOCX) [file pmed.1004045.s026.docx]

**S1 Table. Characteristics of people included in this study from the HKDSD (2001-2019, N=2,608,973)**

|  | N | % |
| --- | --- | --- |
| Sex |  |  |
| Women | 1,413,662 | 54.2% |
| Men | 1,195,311 | 45.8% |
| People who never be identified as prediabetes or diabetes | 1,013,483 | 38.9% |
| People who had ever been identified as prediabetes | 1,014,795 | 38.9% |
| People who had ever been identified as diabetes | 830,870 | 31.9% |
| People who had ever been identified as prediabetes and diabetes | 250,175 | 9.6% |
| Stratified by onset age of prediabetes |  |  |
| <20 years | 5,498 | 0.5% |
| 20-39 years | 47,125 | 4.6% |
| 40-59 years | 382,781 | 37.7% |
| >=60 years | 579,391 | 57.1% |
| Stratified by onset age of diabetes |  |  |
| <20 years | 2,549 | 0.3% |
| 20-39 years | 30,026 | 3.6% |
| 40-59 years | 300,204 | 36.1% |
| >=60 years | 498,091 | 60.0% |
