# Supplementary material for: Lifetime risk of developing diabetes in Chinese people with normoglycemia or prediabetes: A modeling study
Source: PLoS Med. 2022 Jul 21;19(7):e1004045. doi: 10.1371/journal.pmed.1004045 (PMC9302798; doi:10.1371/journal.pmed.1004045)
Supplement: S2 Table — HKDSD, Hong Kong Diabetes Surveillance Database. (DOCX) [file pmed.1004045.s027.docx]

**S2 Table. Clinical characteristics of people included in this study from the HKDSD at baseline.**

|  | Value |
| --- | --- |
| Number | 2,608,973 |
| Age, median (IQR), years | 57.0 (47.0, 68.0) |
| Men, n (%) | 1,195,311 (45.8) |
| Total cholesterol, median (IQR), mmol/L | 5.1 (4.4, 5.8) |
| LDL-C, median (IQR), mmol/L | 3.1 (2.5, 3.7) |
| Triglyceride/HDL-C, median (IQR) | 0.9 (0.6, 1.5) |
| Haemoglobin, median (IQR), g/dL | 13.6 (12.5, 14.6) |
| Albumin, median (IQR), g/L | 42.0 (39.5, 45.0) |
| History of CVD, n (%) ^*^ | 195,345 (7.5) |
| History of CKD, n (%) ^*^ | 149,615 (5.7) |
| History of CHF, n (%) ^*^ | 39,226 (1.5) |
| History of cancer, n (%) ^*^ | 73,157 (2.8) |
| Use of blood pressure medications, n (%) | 2,608,973 (100.0) |
| Use of lipid-regulating drugs, n (%) | 257,899 (9.9) |
| Charlson morbidity index, median (IQR) | 0.0 (0.0, 1.0) |

^*^: History of a disease refers to a person met criteria for these morbidities from 1st January 2000 until baseline. CHF: Congestive heart failure; CKD: chronic kidney disease; CVD: cardiovascular disease; HDL-C: high-density lipoprotein cholesterol; IQR: inter-quantile range; LDL-C: low-density lipoprotein cholesterol.
