# Supplementary material for: Lifetime risk of developing diabetes in Chinese people with normoglycemia or prediabetes: A modeling study
Source: PLoS Med. 2022 Jul 21;19(7):e1004045. doi: 10.1371/journal.pmed.1004045 (PMC9302798; doi:10.1371/journal.pmed.1004045)
Supplement: S3 Table — (DOCX) [file pmed.1004045.s028.docx]

**S3 Table. Characteristics of simulated cohort (N=3,000,000).**

|  | N | % |
| --- | --- | --- |
| Sex |  |  |
| Women | 1,627,819 | 54.3% |
| Men | 1,372,181 | 45.7% |
| People who never be identified as prediabetes or diabetes | 267,965 | 8.9% |
| People who had ever been identified as prediabetes | 1,743,856 | 58.1% |
| People who had ever been identified as diabetes | 1,979,219 | 66.0% |
| People who had ever been identified as prediabetes and diabetes | 991,040 | 33.0% |
| Stratified by onset age of prediabetes |  |  |
| <20 years | 6,400 | 0.4% |
| 20-39 years | 56,272 | 3.2% |
| 40-59 years | 523,206 | 30.0% |
| >=60 years | 1,157,978 | 66.4% |
| Stratified by onset age of diabetes |  |  |
| <20 years | 2,923 | 0.2% |
| 20-39 years | 39,931 | 2.0% |
| 40-59 years | 467,921 | 23.6% |
| >=60 years | 1,468,444 | 74.2% |
