# Supplementary material for: Lifetime risk of developing diabetes in Chinese people with normoglycemia or prediabetes: A modeling study
Source: PLoS Med. 2022 Jul 21;19(7):e1004045. doi: 10.1371/journal.pmed.1004045 (PMC9302798; doi:10.1371/journal.pmed.1004045)
Supplement: S4 Table — (DOCX) [file pmed.1004045.s029.docx]

**S4 Table. Expected remaining life years with diabetes for people with prediabetes and normoglycaemia at age 20, 40, and 60 years estimated in primary analyses and sensitivity analyses.**

|  | All people | | Men | | Women | |
| --- | --- | --- | --- | --- | --- | --- |
|  | From prediabetes | From normoglycaemia | From prediabetes | From normoglycaemia | From prediabetes | From normoglycaemia |
| **Estimates in the primary results** | | | | | | |
| Age 20 years | 32.5 (32.0, 33.1) | 12.7 (12.7, 12.7) | 30.3 (29.7, 31.0) | 12.0 (11.9, 12.0) | 35.8 (35.0, 36.7) | 13.3 (13.2, 13.3) |
| Age 40 years | 24.3 (24.1, 24.4) | 12.0 (12.0, 12.0) | 22.3 (22.1, 22.5) | 11.2 (11.2, 11.3) | 26.1 (25.9, 26.3) | 12.6 (12.6, 12.6) |
| Age 60 years | 12.1 (12.0, 12.1) | 7.8 (7.8, 7.9) | 10.3 (10.2, 10.3) | 7.1 (7.1, 7.1) | 13.5 (13.4, 13.5) | 8.4 (8.4, 8.5) |
| **Simulation from age 20 years onwards** | | | | | | |
| Age 21 years^*^ | 32.5 (30.8, 34.2) | 12.7 (12.7, 12.7) | 30.6 (28.4, 32.7) | 12.0 (12.0, 12.0) | 34.7 (32.1, 37.2) | 13.3 (13.2, 13.3) |
| Age 40 years | 24.4 (24.2, 24.5) | 12.0 (12.0, 12.0) | 22.4 (22.2, 22.6) | 11.2 (11.2, 11.3) | 26.1 (25.9, 26.3) | 12.6 (12.6, 12.6) |
| Age 60 years | 12.0 (12.0, 12.1) | 7.9 (7.9, 7.9) | 10.3 (10.2, 10.3) | 7.1 (7.1, 7.1) | 13.4 (13.4, 13.5) | 8.5 (8.4, 8.5) |
| **Prediabetes was defined by FPG only** | | | | | | |
| Age 20 years | 35.4 (34.6, 36.1) | 12.6 (12.5, 12.6) | 33.3 (32.3, 34.2) | 11.9 (11.9, 11.9) | 38.7 (37.5, 39.9) | 13.1 (13.1, 13.1) |
| Age 40 years | 26.3 (26.1, 26.4) | 11.9 (11.9, 11.9) | 23.8 (23.6, 24.0) | 11.2 (11.2, 11.3) | 28.9 (28.7, 29.2) | 12.5 (12.5, 12.5) |
| Age 60 years | 13.1 (13.1, 13.2) | 7.9 (7.9, 7.9) | 11.2 (11.1, 11.2) | 7.1 (7.1, 7.2) | 14.8 (14.7, 14.8) | 8.5 (8.4, 8.5) |
| **Prediabetes was defined by HbA1c only** | | | | | | |
| Age 20 years | 34.3 (33.6, 34.9) | 12.4 (12.4, 12.4) | 32.3 (31.5, 33.2) | 11.8 (11.8, 11.8) | 37.3 (36.2, 38.3) | 12.9 (12.9, 13.0) |
| Age 40 years | 25.1 (24.9, 25.3) | 11.8 (11.8, 11.8) | 23.3 (23.1, 23.6) | 11.2 (11.2, 11.2) | 26.8 (26.5, 27.0) | 12.4 (12.3, 12.4) |
| Age 60 years | 12.1 (12.0, 12.1) | 8.0 (8.0, 8.0) | 10.5 (10.4, 10.6) | 7.2 (7.2, 7.3) | 13.3 (13.2, 13.4) | 8.6 (8.6, 8.6) |
| **The Sullivan life table method** | | | | | | |
| Age 20 years | 32.7 (32.7, 32.8) | 11.5 (11.5, 11.5) | 31.1 (31.0, 31.1) | 11.2 (11.2, 11.2) | 34.7 (34.6, 34.7) | 11.7 (11.7, 11.7) |
| Age 40 years | 25.2 (25.2, 25.2) | 11.5 (11.4, 11.5) | 23.9 (23.9, 23.9) | 11.1 (11.1, 11.1) | 26.7 (26.7, 26.7) | 11.7 (11.7, 11.7) |
| Age 60 years | 16.2 (16.2, 16.2) | 10.3 (10.3, 10.3) | 14.5 (14.5, 14.5) | 9.7 (9.7, 9.7) | 18.1 (18.1, 18.1) | 10.7 (10.7, 10.7) |

*: People were all assumed to have normoglycaemia at age 20 years when we simulated from age 20 years onward, there was no people with prediabetes at age 20 years, so here we provided estimations for age 21 years.
