# Supplementary material for: Lifetime risk of developing diabetes in Chinese people with normoglycemia or prediabetes: A modeling study
Source: PLoS Med. 2022 Jul 21;19(7):e1004045. doi: 10.1371/journal.pmed.1004045 (PMC9302798; doi:10.1371/journal.pmed.1004045)
Supplement: S7 Table — CHARLS, China Health and Retirement Longitudinal Survey; HKDSD, Hong Kong Diabetes Surveillance Database. (DOCX) [file pmed.1004045.s032.docx]

**S7 Table. Expected remaining lifetime risks of progression from prediabetes and normoglycaemia to diabetes, expressed as percentages, at age 45 years estimated in CHARLS sample, HKDSD sample, and HKDSD sample with mortality rates substituted.**

|  | From prediabetes | From normoglycaemia |
| --- | --- | --- |
| CHARLS | 75.9 (73.0, 78.8) | 54.8 (54.7, 54.8) |
| HKDSD | 81.6 (81.1, 82.1) | 58.5 (58.4, 58.5) |
| HKDSD with mortality rates substituted | 78.9 (78.3, 79.5) | 54.8 (54.8, 54.9) |
| Difference between HKDSD and CHARLS | 5.7 (5.6, 5.8) | 3.7 (3.6, 3.8) |
| Difference between HKDSD with mortality rates substituted and CHARLS | 3.0 (2.9, 3.1) | 0.1 (0.0, 0.1) |
